# Hepatic deletion of *Mboat7* (LPIAT1) causes activation of SREBP-1c and fatty liver

**DOI:** 10.1194/jlr.RA120000856

**Published:** 2021-02-06

**Authors:** Mingfeng Xia, Preethi Chandrasekaran, Shunxing Rong, Xiaorong Fu, Matthew A. Mitsche

**Affiliations:** 1 Center for Human Nutrition, University of Texas Southwestern Medical Center, Dallas, TX, USA; 2 Department of Endocrinology and Metabolism, Zhongshan Hospital, Fudan University, Shanghai, China; 3 Department of Molecular Genetics, University of Texas Southwestern Medical Center, Dallas, TX, USA

**Keywords:** fatty liver disease, phosphatidylinositol, obesity, lipidomics, flux analysis, membrane-bound *O*-acyltransferase domain-containing 7, lysophosphatidylinositol acyltransferase 1, sterol regulatory element-binding protein 1c, liver-specific knockout, AA, arachidonic acid, ACC, acetyl-CoA carboxylase, ACL, ATP citrate lyase, AKT, protein kinase B, ASO, anti-sense oligonucleotide, DAG, diacylglyceride, dKO, double KO, DNL, de novo lipogenesis, GTT, glucose tolerance test, IRS, insulin receptor substrate, ITT, insulin tolerance test, LPIAT, lysophosphatidylinositol acyltransferase, LPLAT, lysophospholipid acyltransferase, MBOAT7, membrane-bound *O*-acyltransferase domain-containing 7, MRM, multiple reaction monitoring, mTOR, mammalian target of rapamycin, NAFLD, non-alcoholic fatty liver disease, PC, phosphatidylcholine, PE, phosphatidylethanolamine, PG, phosphatidylglycerol, PI, phosphatidylinositol, PNPLA3, patatin-like phospholipase domain-containing 3, Scap, SREBP cleavage-activating protein, TMC4, transmembrane channel 4, TM6sf2, transmembrane 6 superfamily member 2, TG, triglyceride

## Abstract

Genetic variants that increase the risk of fatty liver disease and cirrhosis have recently been identified in the proximity of membrane-bound *O*-acyltransferase domain-containing 7 (*MBOAT7*). To elucidate the link between these variants and fatty liver disease, we characterized *Mboat7* liver-specific KO mice (*Mboat7* LSKO). Chow-fed *Mboat7* LSKO mice developed fatty livers and associated liver injury. Lipidomic analysis of liver using MS revealed a pronounced reduction in 20-carbon PUFA content in phosphatidylinositols (PIs) but not in other phospholipids. The change in fatty acid composition of PIs in these mice was associated with a marked increase in de novo lipogenesis because of activation of SREBP-1c, a transcription factor that coordinates the activation of genes encoding enzymes in the fatty acid biosynthesis pathway. Hepatic removal of both SREBP cleavage-activating protein (*Scap*) and *Mboat7* normalized hepatic triglycerides relative to *Scap*-only hepatic KO, showing that increased SREBP-1c processing is required for *Mboat7*-induced steatosis. This study reveals a clear relationship between PI fatty acid composition and regulation of hepatic fat synthesis and delineates the mechanism by which mutations in MBOAT7 cause hepatic steatosis.

## Graphical abstract

**Figure:**
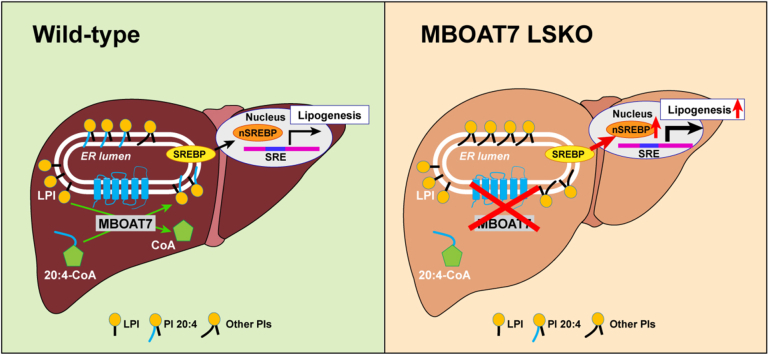

Non-alcoholic fatty liver disease (NAFLD) is the leading cause of chronic liver disease with a global prevalence of ∼24% (1). NAFLD, especially its more severe form of non-alcoholic steatohepatitis, can progress to advanced fibrosis, cirrhosis, hepatocellular carcinoma, and liver-related mortality (2). NAFLD has recently overtaken hepatitis C as the most common risk factor for hepatocellular carcinoma (3) and the second most common indication for liver transplantation in the US (2). The ever-increasing frequency of NAFLD is due to the burgeoning prevalence of obesity and insulin resistance (4), the two most important risk factors for NAFLD. Genetic factors also contribute importantly to the development of this disorder (5), the two most important of which are missense variants in patatin-like phospholipase domain-containing 3 (PNPLA3)-I148M (6, 7, 8, 9) and in transmembrane 6 superfamily member 2 (TM6sf2) (7, 10).

A new risk locus was recently identified that was captured in a genome-wide association study by SNPs located in the first exon of transmembrane channel 4 (*TMC4*) and in the intergenic region between *TMC4* and membrane-bound *O*-acyltransferase domain-containing 7 (*MBOAT7*) gene (11, 12, 13). Despite the proximity of the SNPs to *TMC4*, it is unlikely that *TMC4* directly causes NAFLD because this gene is expressed at very low levels in liver (12), and *Tmc4* KO mice do not exhibit fatty liver (14). In contrast, *MBOAT7* is expressed at high levels in liver, and the mRNA and protein levels of MBOAT7 are decreased in livers of patients with the risk SNPs. Plasma lipidomics analysis has shown a decrease in PI38:4, the primary product of MBOAT7, in patients with the risk SNPs, suggesting that it is a loss-of-function variant (11).

MBOAT7, also known as lysophosphatidylinositol acyltransferase (LPIAT) 1, is a 472-amino acid protein with six transmembrane domains that shares closest sequence similarity to MBOAT5 (LPCAT3) (15). Inactivation of hepatic MBOAT5 in mice causes NAFLD (16). Both MBOAT5 and MBOAT7 have lysophospholipid acyltransferase (LPLAT) activity where they incorporate Ω-6 fatty acids into the sn-2 position of lysophospholipids as part of the Land's cycle when expressed in yeast (17). The two enzymes differ, however, in fatty acid substrate specificities. MBOAT5 incorporates arachidonic acid (AA) or linoleic acid into phosphatidylcholine (PC) or phosphatidylethanolamine (PE), whereas MBOAT7 incorporates AA into phosphatidylinositol (PI) (17). The individuals with the risk SNPs at the *TMC4*/*MBOAT7* locus have a decrease in the amount of AA in circulating PIs, which further supports the notion that MBOAT7 activity is deficient in these patients (11).

Two previous studies showed that germline *Mboat7* KO mice die within 1 month of birth from defective brain development (18, 19) accompanied by reduced levels of arachidonoyl-PIs in all surveyed tissues (18, 19). The phenotype of the KO mice resembles that seen in humans with genetic deficiency in *MBOAT7* (20). To determine whether MBOAT7 deficiency is the cause of the genetic association of the *TMC4-MBOAT7* locus with NAFLD, we developed mice expressing no *Mboat7* in the liver. Here, we show that these mice have increased hepatic triglyceride (TG) content. By performing detailed molecular characterization in the livers of these mice, we also determined the mechanism responsible for the increase in hepatic fat in these animals, which was correlated with the processing of liver SREBP-1c.

## Materials and Methods

### Animals

Mice harboring *Mboat7* floxed alleles were purchased from the International Mouse Phenotyping Consortium (https://www.mousephenotype.org/data/genes/MGI:1924832). CRISPR-Cas9 technology was used to insert one *lox*P site downstream of exon 5 and another *lox*P site upstream of exon 5 of *Mboat7*. A *Lac*Z trapping cassette was placed 5′ of a *lox*P-flanked promoter-driven neomycin-resistance selection cassette in the gene targeting construct. The *Lac*Z trapping cassette and neomycin-resistance selection cassette are flanked by two flippase recognition target sites. The heterozygous *Mboat7* floxed mice with the *lac*Z and neomycin-resistance cassettes were crossed with mice expressing FLP1 recombinase to excise the flippase recognition target-flanked sites and create heterozygous *Mboat7* floxed (*Mboat7*^*f*/+^) mice. *Mboat7*^*f*/+^ mice were then crossed with *Mboat7*^*f*/+^ mice to generate homozygous *Mboat7*^*f/f*^ mice. Further crossing of homozygous *Mboat7*^*f/f*^ mice with Alb-Cre mice of C57BL/6J background generated *Mboat7*^*f*/+^ mice with Alb-Cre transgene (*Mboat7* LSKO) and *Mboat7*^*f/f*^ mice without Alb-Cre transgene (WT) (supplemental Fig. S1A). Homozygous *Mboat7*^*f/f*^ mice were bred with *Scap*^*f*/+^ to generate *Mboat7/Scap*^*f*/+^ mice. These mice were bred to generate *Mboat7*^*f/f*^*/Scap*^*f/f*^ and crossed with Alb-Cre transgenic mice to generate liver-specific double KO (dKO) of *Mboat7* and *Scap* (*Mboat7/Scap* dKO) mice.

### Diet studies

All experiments shown were performed using 12- to 14-week-old male mice on a C57BL/6J background (Jackson Laboratory) unless noted otherwise. Duplicate studies were carried out using female mice with similar results. Mice were maintained on a 12 h light/12 h dark cycle and fed Teklad 2016 chow diet with free access to water. For all experiments, chow-fed mice were fasted for 16 h (5:00 PM to 9:00 AM) and refed chow for 8 h (9:00 AM to 5:00 PM) for 3 days. On the morning of the fourth day, mice were refed for 4 h (9:00 AM to 1:00 PM) and euthanized. This fasting-refeeding regime was utilized to study the mice when SREBPs were at maximum activation. Plasma, liver, and kidney tissues were collected and frozen at −80°C, and body weights and liver weights were recorded. All experiments were repeated under similar conditions. All experimental protocols and procedures were approved by the University of Texas Southwestern Medical Center Institutional Animal Care and Use Committee.

### Plasma biochemical measurements

Blood was drawn from the inferior vena cava, and plasma was separated immediately by centrifuging whole blood in an EDTA tube at 10,000 *g* for 5 min and storing at −80°C. Plasma concentrations of cholesterol, TGs, glucose, alanine aminotransferase, and aspartate aminotransferase were measured using VITROS 250 Microslide Technology by the University of Texas Southwestern Metabolic Phenotyping Core (https://utsw.corefacilities.org/landing/170). Plasma insulin was measured using an ultra-sensitive mouse insulin ELISA kit (Crystal Chem). Total liver lipids were extracted, and liver cholesterol and TG contents were measured using enzymatic assays (Infinity, Thermo Electron Corp., and Wako Inc.) by the University of Texas Southwestern Metabolic Phenotyping Core.

### Histopathology analysis

A small piece of fresh liver tissue (∼300 mg) was fixed in 10% neutral buffered formalin (Sigma-Aldrich) at 4°C for 24 h with gentle shaking and sent to the University of Texas Southwestern Histo Pathology Core (http://utsouthwestern.edu/labs/histo-pathology) for hematoxylin-eosin staining and Masson trichrome staining.

### Quantitative real-time PCR

Total RNA was extracted from mouse livers using RNA STAT-60 (Tel-Test). cDNA was synthesized from 2 μg of DNase I-treated total RNA (DNA-free, DNA removal kit; Invitrogen) using TaqMan reverse transcription reagents (Applied Biosystems, Carlsbad, CA) and random hexamer primers. The real-time RT-PCR reaction included 20 ng of reverse-transcribed total RNA, 167 nM of the forward and reverse primers, and 10 μl of 2× SYBR Green PCR Master Mix (Applied Biosystems). PCR reactions were carried out in 384-well plates using the ABI PRISM 7900HT sequence detection system (Applied Biosystems). All reactions were done in triplicate, and the relative amount of all mRNAs was calculated using the comparative threshold cycle method. Cyclophilin mRNA was used as invariant control. The primers for real-time PCR are listed in the supplemental Table S1.

### Immunoblot analysis

Liver samples (∼100 mg) from snap-frozen mouse liver tissue were homogenized using 1 ml of buffer A [1% SDS, 1% Triton X-100%, 1% NP-40, 50 mM Tris-HCl (pH 7.4), 50 mM EDTA] containing a mixture of protease inhibitors (2 mM PMSF, 50 μg/ml ALLN, 20 μg/ml aprotinin, 50 μg/ml leupeptin, and one tablet of Roche protease inhibitor per 10 ml). Equal aliquots of protein from individual livers within each group were pooled (total, 40 μg), and proteins were subjected to 8% SDS-PAGE gels and transferred to nitrocellulose membrane (Bio-Rad, Hercules, CA). Immunoblot analyses were performed using SREBP-1, SREBP-2, FAS, acetyl-CoA carboxylase (ACC)1, ACC2, ATP citrate lyase (ACL), insulin receptor substrate (IRS)1, phospho-IRS1, IRS2, phospho-IRS2, protein kinase B (AKT), phospho-AKT(Ser473), phospho-AKT(Thr308), mammalian target of rapamycin (mTOR), phospho-mTOR, S6 ribosomal protein, and phosphor-S6 ribosomal protein antibodies listed in supplemental Table S1. SuperSignal West Pico chemiluminescent substrate system (Thermo Scientific) was used to detect the antibody-bound bands. Anti-CREB and anti-β-actin antibodies were used as loading controls for the proteins, respectively.

### In vivo VLDL secretion measurements

Mice were fasted for 4 h, and 10% Triton WR-1339/saline solution (Tyloxapol; Sigma-Aldrich) (500 mg/kg) was injected into mice intravenously. Blood was collected from the tail vein 0, 0.5, 1, 1.5, 2, 2.5, and 3 h after the Triton injection, and plasma was separated for measurement of TG levels. The plasma TG secretion rates were calculated from the slope of the regression line of the time versus TG concentration.

### Glucose and insulin tolerance test

A glucose tolerance test (GTT) was performed on overnight-fasted mice, and 20% glucose solution (1 mg/g body weight) was injected into mice intraperitoneally. For the insulin tolerance test (ITT), mice were fasted for 4 h followed by an intraperitoneal injection of insulin (1 mU/g for male and 0.7 mU/g for female). Blood was collected from the tail vein 0, 15, 30, 60, and 120 min after the injection of glucose or insulin and immediately tested for blood glucose using a glucose meter (Bayer contour glucose meter; Bayer).

### Liver tissue ER membrane fraction

ER membrane isolation from liver was obtained using a modified cell fractionation protocol developed by Radhakrishnan *et al.* (21). Mice were euthanized after 4 h refeeding between 10:00 AM and 11:00 AM. Fresh liver tissue (500 mg per mouse) was homogenized in ice-cold buffer B [50 mM Tris-HCl (pH 7.5), 150 mM NaCl] containing 15% (w/v) sucrose using a 2 ml dounce homogenizer (eight strokes). The homogenized whole-liver tissue (fraction A) was centrifuged at 15,000 *g* for 10 min to yield a pellet (fraction B) and a supernatant. The supernatant was centrifuged at 15,000 *g* for 10 min, a second time to collect the supernatant (fraction C). Fraction C was diluted to a total volume of 3 ml using buffer B containing 15% sucrose. A discontinuous sucrose gradient (sucrose gradient 1) was generated in a Beckman centrifuge tube (Beckman, #344059) by overlaying the following sucrose solutions in buffer B: 2 ml 45%, 4 ml 30%, 3 ml of the diluted supernatant in 15% sucrose, and 2 ml 7.5% of sucrose buffer B. The gradient was centrifuged at 100,000 *g* in a TH641 rotor for 1 h and allowed to slow without the application of a brake, after which two bands of membranes were clearly visible and collected using a Pasteur pipette. The light membrane was collected (fraction D), and the heavy membrane (fraction E) was then diluted to a total volume of 3 ml using buffer B containing 45% sucrose. A second discontinuous sucrose gradient (sucrose gradient 2) was generated by overlaying 2 ml 60%, 4 ml 51%, 3 ml of the diluted fraction E in 45% sucrose, and 2 ml of 30% sucrose buffer B solution. The second gradient was centrifuged at 110,000 *g* for 70 min to further remove cellular mitochondria from fraction E. A discontinuous iodixanol gradient was generated by underlaying, in succession, 2.25 ml of 19%, 21%, 23%, and 25% (v/v) iodixanol, all in buffer B. This discontinuous gradient was allowed to stand for 1 h, allowing for diffusion across the interfaces to form a continuous gradient. The light membrane between the 30% and 45% sucrose layer in sucrose gradient 2 was collected and loaded at the bottom of continuous iodixanol gradient, which was then centrifuged for 2 h at 100,000 *g* in a TH641 rotor and allowed to slow without the application of a brake. Nine fractions (1 ml each) were collected from the bottom. The heavier fractions from tubes 2–6 were pooled as pure ER fraction (fraction F). Fraction F was then divided into two portions: one portion was concentrated in 20 μl buffer B for protein concentration measurement using a BCA kit (Pierce), and a second portion was used for lipid composition analysis described below.

### Liver membrane fraction preparation

For liver membrane fraction preparation, aliquots of frozen liver tissue (50–70 mg) were homogenized in 1 ml of buffer C [20 mM Tris-HCl (pH 7.4), 2 mM MgCl_2_, 250 mM sucrose, 10 mM EDTA, 10 mM EGTA] with a mixture of protease inhibitors (2 mM PMSF, 50 μg/ml ALLN, 20 μg/ml aprotinin, 50 μg/ml leupeptin, 1 mM DTT, and one tablet of Roche protease inhibitor per 10 ml) using a physcotron homogenizer (Microtec). Homogenized samples were then centrifuged at 800 *g* for 10 min. The supernatants were centrifuged at 100,000 *g* for 1 h. Pellets were resuspended in buffer D [20 mM Tris-HCl (pH 7.4), 300 mM sucrose, 1 mM EDTA], and protein concentrations were measured using a BCA kit.

### LPLAT activity assay

LPLAT activity was measured as previously described (17). In short, LPLAT activity was measured by incubating membrane fractions (10 μg of membrane protein) with a mix of substrates at 37°C for 10 min. Substrate mixtures contained 3 μM deuterium-labeled 16:0 LPC, 17:1 LPE, 17:1 LPG, 17:1 LPI, and 3 μM each of 16:0-, 18:0-, 18:1-, 18:2-, 18:3-, 20:4-, and 22:6-CoA (Avanti). The reaction buffer contained 110 mM Tris-HCl (pH 7.4), 150 mM sucrose, and 0.5 mM EDTA. Reactions were stopped by the addition of methanol. LipidoMix™ internal standard mixture (Avanti) was added before Bligh and Dryer extraction to normalize extraction efficiency. Reaction products were quantified using LC-MS/MS (see below) by comparing peak areas of chromatograms to those of lipid standards.

### LC-MS/MS phospholipid analysis

Approximately 20 mg of tissue was weighed and homogenized in 1 ml of methanol in 2.0 ml prefilled Bead Ruptor tubes (2.8 mm ceramic beads; Omni International, Kennesaw, GA). The tubes were washed twice with 1 ml of methanol. To the homogenates, 10 μl of 1:1,000 diluted SPLASH LipidoMix™ internal standard was added followed by the modified Bligh and Dyer extraction with DCM/methanol/saline 2:1:1 (22). After centrifugation, the lipid phase at the bottom was isolated, dried under a stream of nitrogen, and redissolved in methanol before being subjected to LC-MS/MS analysis described below.

The isolated lipid samples were analyzed using a Nexera UHPLC system (Shimadzu) coupled to a QTRAP 4000 mass spectrometer (AB Sciex). LC was performed on an ACE HILIC-A column (150 × 2.1 mm, 3 μm; Advanced Chromatography Technologies Limited). The SPLASH LipidoMix™ standard and lipids mixture was separated using a mobile phase consisting of water/acetonitrile (5:95, v/v) with 5 mM ammonium acetate (eluent A), and water/acetonitrile (50:50, v/v; eluent B) with 5 mM ammonium acetate adjusted to pH 5.5. The flow rate and column temperature were set to 200 μl/min and 25°C, respectively. The optimized gradient started with 0.1% B and then increased to 5% over 5 min. Subsequently, the mobile phase increased to 20% B over 35 min and then increased to 98% B over 10 min, followed by a washing step at 98% B for 10 min and equilibrating for 8 min. The total run time was 70 min. A diverter valve was employed to reduce the introduction of matrix components into the mass spectrometer. Multiple reaction monitoring (MRM) analysis was performed using ESI in negative ion mode with scheduling on QTRAP 4000 MS instrument. The settings of the ESI source were as follows: curtain gas (30 psi), ionization (−4,500 V), temperature (350°C), nebulizer gas (30 psi), and heating gas (40 psi). The mass spectrometer was set to a target scan time of 5 s for the MRM scan survey. The declustering potential, entrance potential, collision energy, and collision cell exit potential for each MRM transition of each lipid class were optimized by using SPLASH LipidoMix™ standard. A total of 354 MRM transitions were monitored for the liver lipids profiling. Intensity values for all targeted lipids were normalized to the intensity of the corresponding standard in the Avanti Splash mixture from the same class.

### Lipid flux analysis

Male mice (12–14 weeks of age) were diet synchronized for 3 days (see above). On the fourth day, the mice were refed for 3 h and then injected with 15 μl/g of deuterium oxide (D_2_O) in 0.7% NaCl (w/w). After 1 h, the mice were euthanized, and liver and plasma were harvested and snap-frozen at −80°C. Samples were prepared for LC-MS/MS phospholipid analysis (see above) and analyzed with a Nexera HPLC system (Shimadzu) coupled to a Fusion Lumos 1M (Thermo Scientific). For TG analysis, lipids were separated on an Agilent Poroshell 120 EC-C18 column (150 × 2.1 mm, 2.7 μm beads; Santa Clara, CA). The TGs were isolated during a 30 min linear gradient transitioning from 100% methanol + 5 mM ammonium acetate to 20% DCM/80% methanol + 5 mM ammonium acetate. Phospholipids were isolated using the protocol described above.

Parallel reaction monitoring analysis targeted the M+1 positive ion isotopes of the pseudo-diacylglyceride (DAG) fragment of TAG containing either 16:0, 16:1, 18:0, or 18:1. For phospholipid analysis, parallel reaction monitoring analysis of the M+1 isotope, which corresponds to the saturated fatty acid fragment of the phospholipids, was performed. The complete isotopic spectrum from M+0 to M+4 of palmitate in PC 36:4(16:0, 20:4) was collected to determine the precursor pool enrichment of deuterium in water in liver using mass isotopomer distribution analysis (23). The number of potential labeling sites for palmitic and palmitoleic acids was assumed to be 22 and for steric and oleic acids was assumed to be 24; the glycerol backbone of the pseudo-DAG fragment was assumed to have six potential labeling sites. Fractional turnover after 1 h was determined using isotopic spectral analysis (24). Briefly, the fractional enrichment (g) was calculated as the measured ratio of D/^13^C (M1m) minus the natural ratio of D/^13^C (M1n) divided by the D/^13^C of a fully labeled molecule (M1t) (based on the precursor enrichment and number of labeling sites) minus M1n:

$$\text{g}=\frac{\text{M}1\text{m}-\text{M}1\text{n}}{\text{M}1\text{t}-\text{M}1\text{n}}$$

Absolute synthesis rates were determined by normalizing the lipid intensity to the standard intensity from the SPLASH mix, multiplying by the fractional turnover value (g), and then dividing by the labeling time (55–70 min).

## Results

### Deletion of *Mboat7* in mouse hepatocytes

To inactivate *Mboat7* selectively in hepatocytes, *lox*P sites were introduced into the introns flanking exon 5 of *Mboat7* using CRISPR-Cas9 technology (supplemental Fig. S1A). Mice were bred to FLP1 transgenic mice to remove the LacZ and neo cassette. Mice homozygous for the *lox*P sites were bred with mice that express Cre recombinase under control of the hepatocyte-specific albumin promoter to generate *Mboat7* LSKO mice. To confirm the deletion of exon 5 in liver, PCR amplification of liver mRNA was performed. The liver *Mboat7* expression was deleted in *Mboat7* LSKO mice (*P* < 0.001), with no differences in *Mboat7* expression in kidney (supplemental Fig. S1B). The adjacent *Tmc4* and closely related *Mboat5* (*Lpcat3*) mRNA levels were not altered in *Mboat7* LSKO livers.

The Mboat7 LSKO mice were born in the expected Mendelian ratios, and their litter sizes did not differ from WT animals.

### Increased TG and cholesterol concentrations in livers from *Mboat7* LSKO mice

**Fig. 1:**
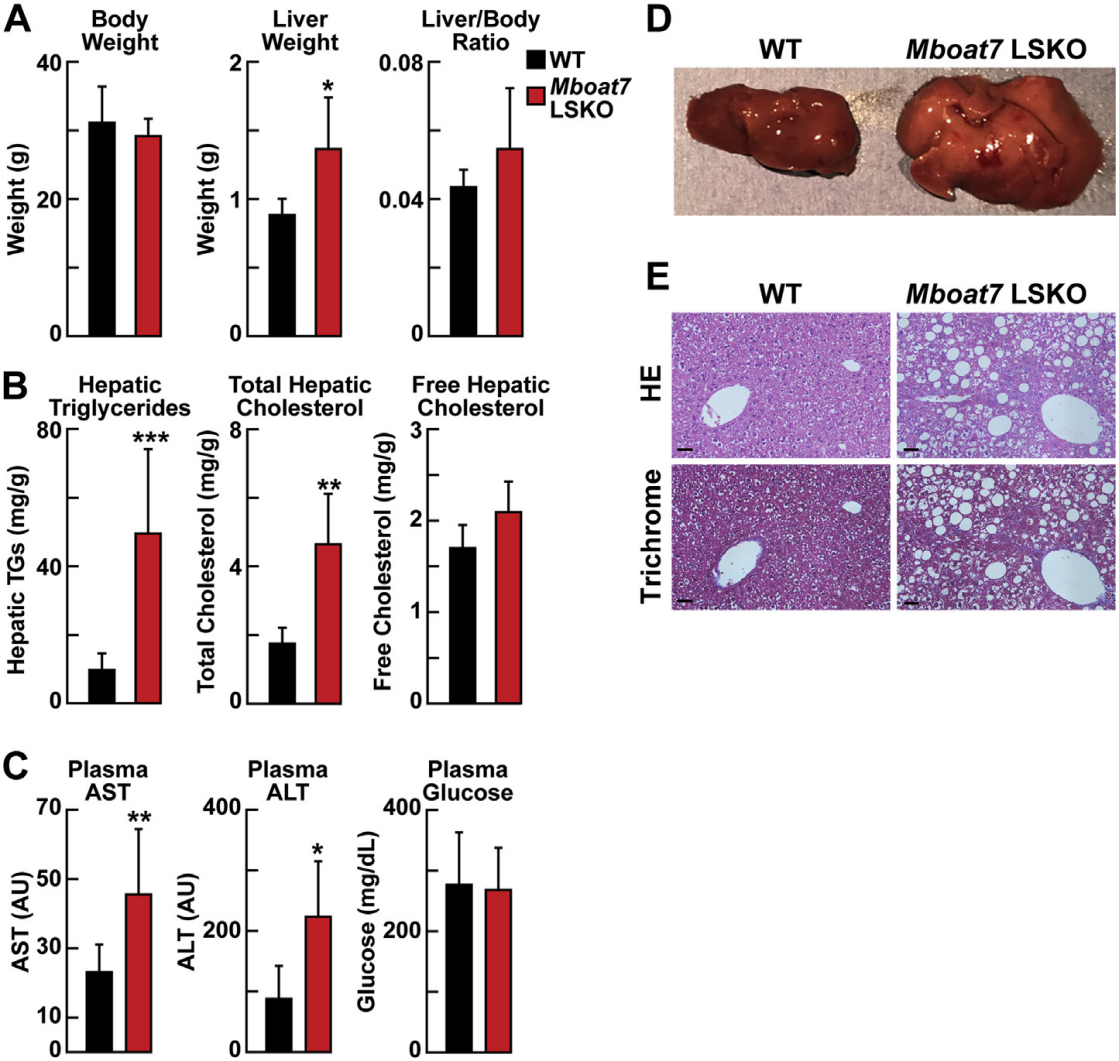
Levels of liver TGs and cholesterol and plasma ALT and AST concentrations in WT and *Mboat7* LSKO mice (n = 5 per group). A: Body weight, liver weight, and liver/body weight ratio in WT and *Mboat7* LSKO mice. B: Representative liver appearance of 1-year-old WT and *Mboat7* LSKO mice fed with a chow diet. Images were taken at the same magnification and distance. C: Liver TGs, total cholesterol, and free cholesterol in WT and *Mboat7* LSKO mice. D: Plasma AST, ALT, and glucose. E: Representative liver HE- and trichrome-stained sections of 1-year-old WT and *Mboat7* LSKO mice fed with a chow diet. Images are taken at 20× magnification. Length bar represents 20 μm. In A–C, each value represents mean ± SD. The asterisks denote the level of statistical significance (Student's *t*-test) between WT and *Mboat7* LSKO mice: ∗*P* < 0.05, ∗∗*P* < 0.005, ∗∗∗*P* < 0.001. ALT, alanine aminotransferase; AST, aspartate aminotransferase; *Mboat7* LSKO, *Mboat7* liver-specific KO mice; TG, triglyceride.

The *Mboat7* LSKO and WT littermates (male, 12 weeks of age) were fasted for 16 h and refed for 4 h before the chow-fed mice were euthanized. *Mboat7* LSKO mice appeared phenotypically normal and had no changes in body weight (Fig. 1A, left panel). The mean liver weight was significantly higher in the KO mice, (Fig. 1A), which was easily discernible on gross inspection of the liver (Fig. 1B). Hepatic TGs and total cholesterol levels were increased approximately fourfold and approximately twofold, respectively, in *Mboat7* LSKO livers when compared with littermate controls. Hepatic-free cholesterol concentrations did not differ between the strains (Fig. 1C). Cholesterol esters compose the majority of nonfree cholesterol and thus likely represent the majority of the total cholesterol increase. Plasma aspartate aminotransferase and alanine aminotransferase levels were also elevated significantly in *Mboat7* LSKO mice, indicating possible liver cell damage (Fig. 1D). Histological hematoxylin-eosin and trichrome staining of liver sections showed marked steatosis in the chow-fed *Mboat7* LSKO mice (Fig. 1E). No differences were measured in plasma glucose levels after a 12 h fast (Fig. 1D).

### Hepatic phospholipid composition is altered in *Mboat7* LSKO mice

**Fig. 2:**
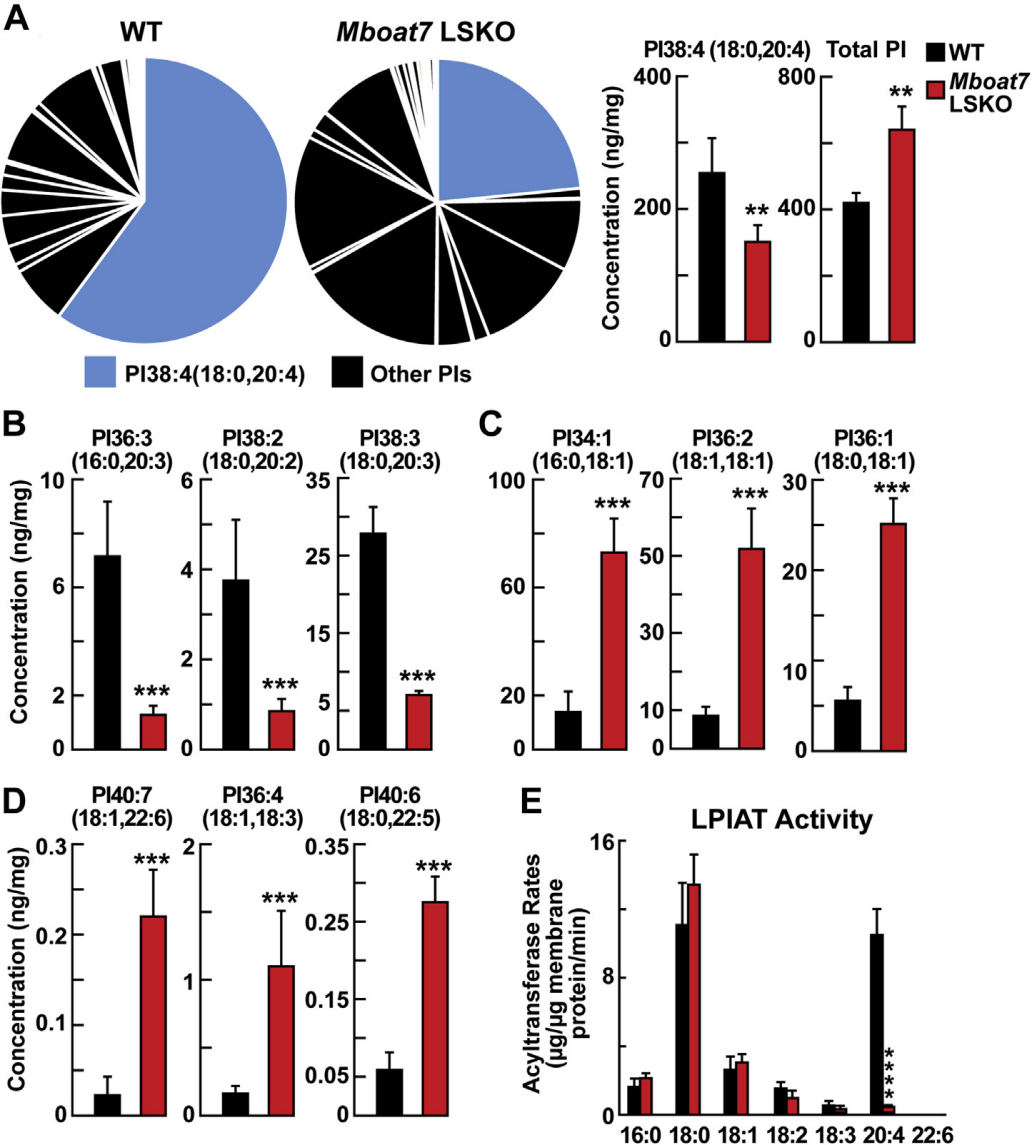
PI comparisons of whole liver from WT and *Mboat7* LSKO mice (n = 6 per group). A: PI composition (left) and concentrations of total PI and PI 38:4(18:0, 20:4) (right) from the whole-liver lysates of WT and *Mboat7* LSKO mice. The percentage of PI 38:4(18:0, 20:4) in total PIs was highlighted in blue color. Hepatic concentrations of PIs that decreased in *Mboat7* LSKO mice with 20-carbon fatty acids PI 36:3(16:0, 20:3), PI 38:2(18:0, 20:2), and PI 38:3(18:0, 20:3) (B); PIs containing saturated and monounsaturated fatty acids PI 34:1(16:0, 18:1), PI 36:2(18:1, 18:1), and PI 36:1(18:0, 18:1) (C); and PIs containing PUFAS PI 40:7(18:1, 22:6), PI 36:4(18:1, 18:3), and PI 40:6(18:0, 22:5) (D) were compared between WT and *Mboat7* LSKO mice. E: LPIAT activity was examined in liver membrane fraction from WT and *Mboat7* LSKO mice (n = 3 per group). The production rates of 16:0-, 18:0-, 18:1-, 18:2-, 18:3-, and 20:4/17:1-PI were compared between WT and *Mboat7* LSKO mice. Data are presented as mean ± SD. The asterisks denote the level of statistical significance (Student's *t*-test) between WT and *Mboat7* LSKO mice: ∗*P* < 0.05, ∗∗*P* < 0.005, ∗∗∗*P* < 0.001, ∗∗∗∗*P* > 0.0005. LPIAT, lysophosphatidylinositol acyltransferase; *Mboat7* LSKO, *Mboat7* liver-specific KO mice; PI, phosphatidylinositol.

Previous studies indicated that *Mboat7* has LPIAT activity that selectively transfers AA into PI. We hypothesized that the marked increase in liver TG and cholesterol contents in *Mboat7* LSKO was likely mediated by the changes in liver phospholipid composition. To test this hypothesis, phospholipid concentrations were measured in the liver by LC-MS/MS. Global analysis of phospholipids in liver lysates revealed no significant differences in the total amount of PC, PE, phosphatidylserine, and phosphatidylglycerol (PG) in WT and *Mboat7* LSKO mice. Striking differences were seen in the PI species. Although the total PI levels in the KO mice were increased, the percentage and concentration of PI 38:4(18:0, 20:4), the predominant PI in liver, was decreased in *Mboat7* LSKO mice by ∼50% (Fig. 2A). This result was not unexpected because PI 38:4(18:0, 20:4) was previously thought to be the primary product for *Mboat7*. Surprisingly, the concentrations of additional PIs with 20-carbon PUFAs, including PI 36:3(16:0, 20:3), PI 38:2(18:0, 20:2), and PI 38:3(18:0, 20:3), were reduced by more than 80% in *Mboat7* LSKO livers compared with WT animals (Fig. 2B). Inactivation of *Mboat7* in the liver had an opposite effect on the levels of PIs with monounsaturated or polyunsaturated PIs without 20-carbon fatty acids, including PI 34:1(16:0, 18:1), PI 36:2(18:1, 18:1), PI 36:1(18:0, 18:1), PI 40:7(18:1, 22:6), PI 36:4(18:1, 18:3), and PI 40:6(18:0, 22:5); these lipids were significantly increased in *Mboat7* LSKO mouse livers (Fig. 2C, D).

LPIAT activity in the liver membrane fractions was then measured ex vivo. The incorporation of 20:4-CoA into PI was markedly reduced in *Mboat7* LSKO mice, accounting for the reduction in PI species containing 20-carbon PUFAs in liver PI composition (Fig. 2E). The incorporation of other fatty acyl-CoAs (16:0-, 18:0-, 18:1-, 18:2-, 18:3-, and 22:6-CoA) into PC, PE, PG, and PI was also tested, and only the incorporation of 20:4-CoA into PI, PC, and PE was significantly altered (supplemental Fig. S2).

### *Mboat7* LSKO does not influence hepatic TG secretion

**Fig. 3:**
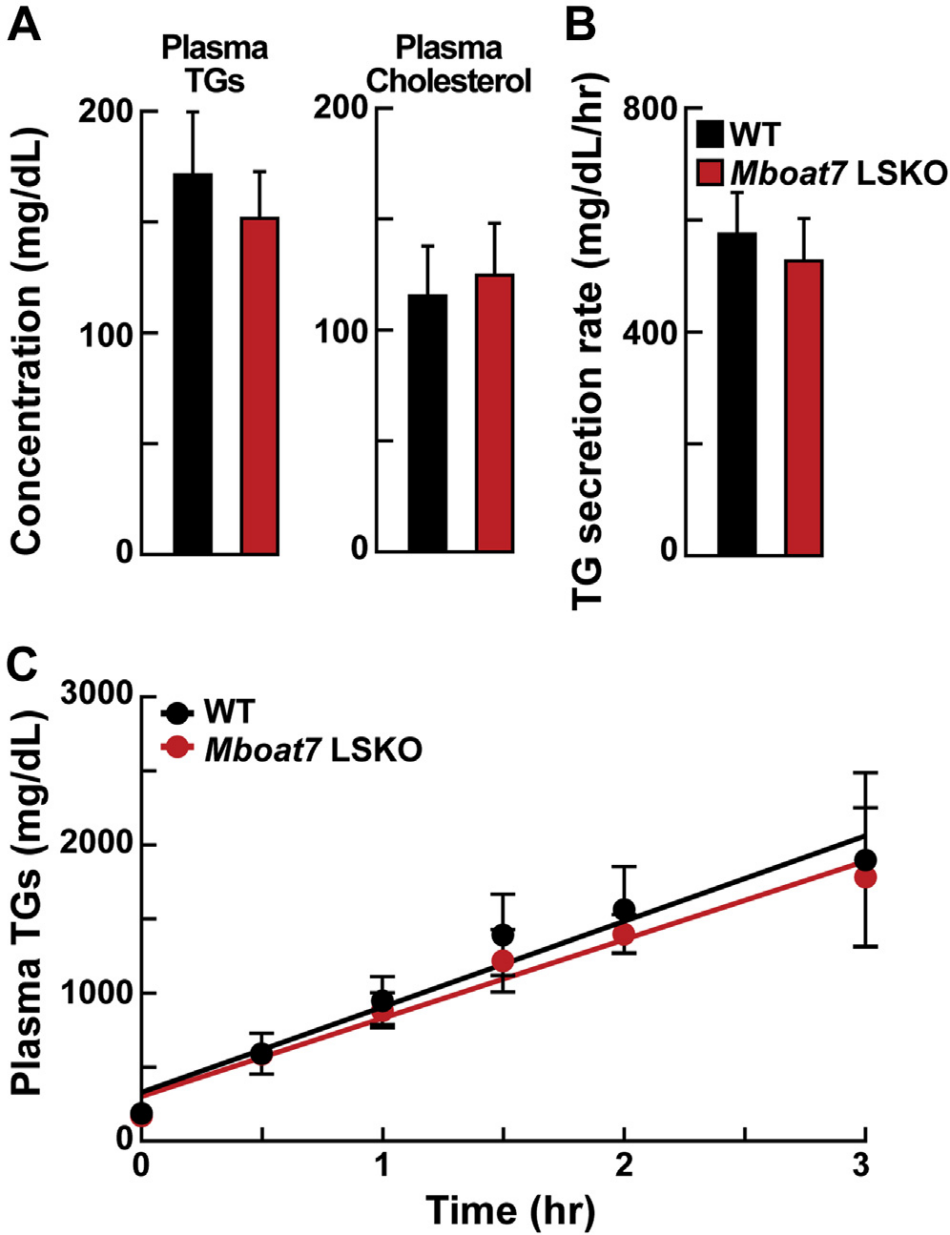
Plasma TG and total cholesterol concentrations and in vivo VLDL secretion in WT and *Mboat7* LSKO mice. A: Plasma TG and cholesterol levels of WT and *Mboat7* LSKO mice (n = 5 per group). B: Plasma TG secretion rate during a detergent block of lipolysis was calculated for each mouse from the linear regression analysis of the time versus TG concentration. C: Six male mice of each genotype were fasted for 4 h before the study. Each mouse was injected intravenously with a 10% Triton-saline solution at 500 mg/kg. Plasma TG accumulation of each mouse at 0, 0.5, 1, 1.5, 2, 2.5, and 3 h after the Triton injection were measured. *Mboat7* LSKO, *Mboat7* liver-specific KO mice; TG, triglyceride.

Previous studies have demonstrated that increased hepatic TGs can result from reduced VLDL secretion, for example in *Tm6sf2* KO mice or *Mboat5* LSKO mice (16, 25, 26). The plasma TG and cholesterol did not differ between WT and *Mboat7* LSKO mice (Fig. 3A). To directly measure hepatic TG secretion, fasting WT and *Mboat7* LSKO mice were injected with Triton WR 1339 to block lipolysis, and TG levels in plasma were measured at various time points. As shown in Fig. 3B, C, TG secretion rates from livers of *Mboat7* LSKO mice were similar to those of WT mice. Therefore, the increase in hepatic TG was not caused by a decrease in VLDL-TG secretion.

### Deletion of *Mboat7* in hepatocytes increases hepatic nuclear SREBP-1c and de novo lipogenesis

**Fig. 4:**
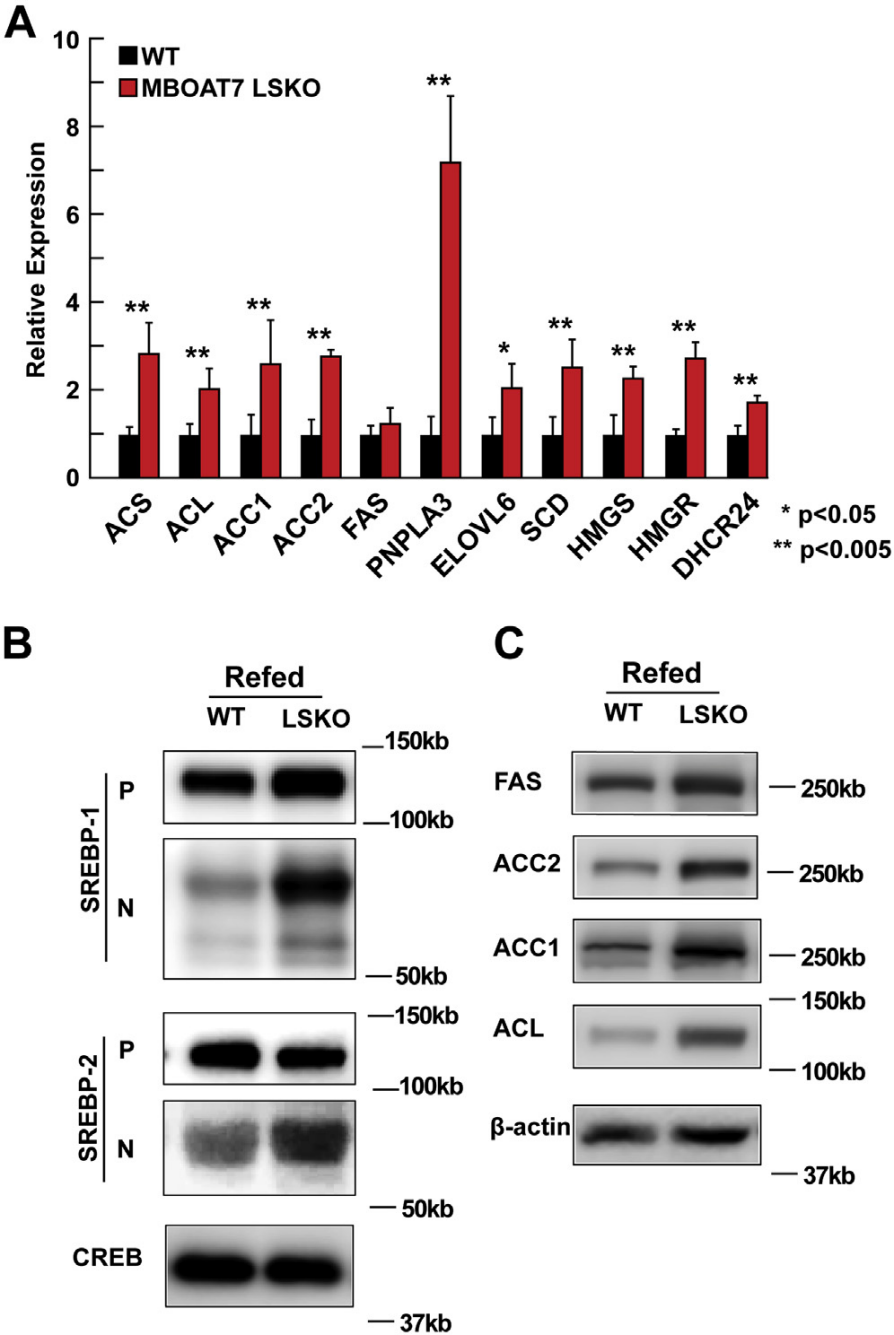
Levels of mRNAs, SREBP, and lipogenic proteins in the livers of WT and *Mboat7*. A: mRNA levels of SREBP-1c and SREBP-2 target genes. Total RNA from each mouse liver was subjected to real-time PCR. Cyclophilin was used as the invariant control. Values represent the amount of mRNA relative to that in the WT mice, which are arbitrarily assigned a value of 1. B: Nuclear SREBP levels were increased in *Mboat7* LSKO livers. Whole-liver lysate was made from each mouse and equal aliquots were pooled (total, 40 μg) and subjected to SDS-PAGE and immunoblot analysis. The precursor and nuclear forms of SREBP-1 and SREBP-2 are denoted as P and N, respectively. C: Immunoblot analysis of SREBP-1c target protein levels responsible for DNL. Data are presented as mean ± SD. The asterisks denote the level of statistical significance (Student's *t*-test) between WT and *Mboat7* LSKO mice: ∗*P* < 0.05, ∗∗*P* < 0.005. *Mboat7* LSKO, *Mboat7* liver-specific KO mice; DNL, de novo lipogenesis.

Increased rates of de novo lipogenesis (DNL) can lead to hepatic steatosis (27, 28). To determine whether inactivation of *Mboat7* in the liver causes hepatic steatosis by increasing the rate of DNL, we examined the mRNA levels of genes encoding enzymes involved in fatty acid and sterol synthesis. As shown in Fig. 4A, hepatic mRNA levels of genes activated by SREBP-1c (*ACS*, *ACL*, *ACC1*, *ACC2*, *PNPLA3*, *ELOVL6*, and *SCD1*) and SREBP-2 (*HMGS*, *HMGR*, and *DHCR24*) were significantly increased in the *Mboat7* LSKO mice. Of the target genes of SREBP-1c, only FAS was not increased in the *MBOAT7* LSKO.

SREBP-1c is a membrane-bound transcription factor that undergoes a series of cleavages that release the transcriptionally active form of the protein (29). Immunoblot analysis of SREBP-1c showed that the active nuclear form of SREBP-1c was increased in *Mboat7* LSKO livers (Fig. 4B). The protein levels of FAS, ACC1, ACC2, and ACL were also increased in liver (Fig. 4C). Similar but smaller increases in the nuclear form of SREBP-2 were detected (Fig. 4B), the SREBP isoform that regulates genes involved in cholesterol biosynthesis (HMGR) and the LDLR.

### Normal glucose homeostasis and insulin signaling in *Mboat7* LSKO mice

SREBP-1c is an insulin-responsive gene that is activated in insulin-resistant states with high levels of circulating insulin (28). To determine whether insulin resistance contributed to the activation of SREBP-1c and increased lipogenesis, we measured plasma glucose and insulin levels in fed mice and found no differences (Fig. 1C, supplemental Fig. S5B). We then carried out a GTT and ITT, and no differences between the two strains were observed (supplemental Fig. S3A). The phosphorylation status of components of the insulin signaling cascade, including IRS1/2, AKT, S6, and mTOR, were also interrogated. Again, no difference was observed in the phosphorylation in these proteins (supplemental Fig. S3C). These results demonstrate that hepatic fat accumulation in *Mboat7* LSKO mice is not driven by altered insulin sensitivity.

### Increased DNL in *Mboat7* LSKO mice

To determine whether the changes in gene expression resulted in changes in rates of DNL, lipid biosynthesis rates were measured by monitoring the rate of deuterium incorporation into lipids by MS. A modification of the assay was introduced into the protocol. We used MS that had sufficient resolving power to discriminate between labeling deuterium isotopomers from naturally occurring ^13^C isotopomers in lipid isotopes. The improvement in signal-to-noise ratio using this method allowed for measurement of the rates of biosynthesis of specific lipid species using a shorter labeling time. Mice were fasted for 12 h and then refed for 3 h before D_2_O was administered by intraperitoneal injection. The mice were euthanized 1 h later.

**Fig. 5:**
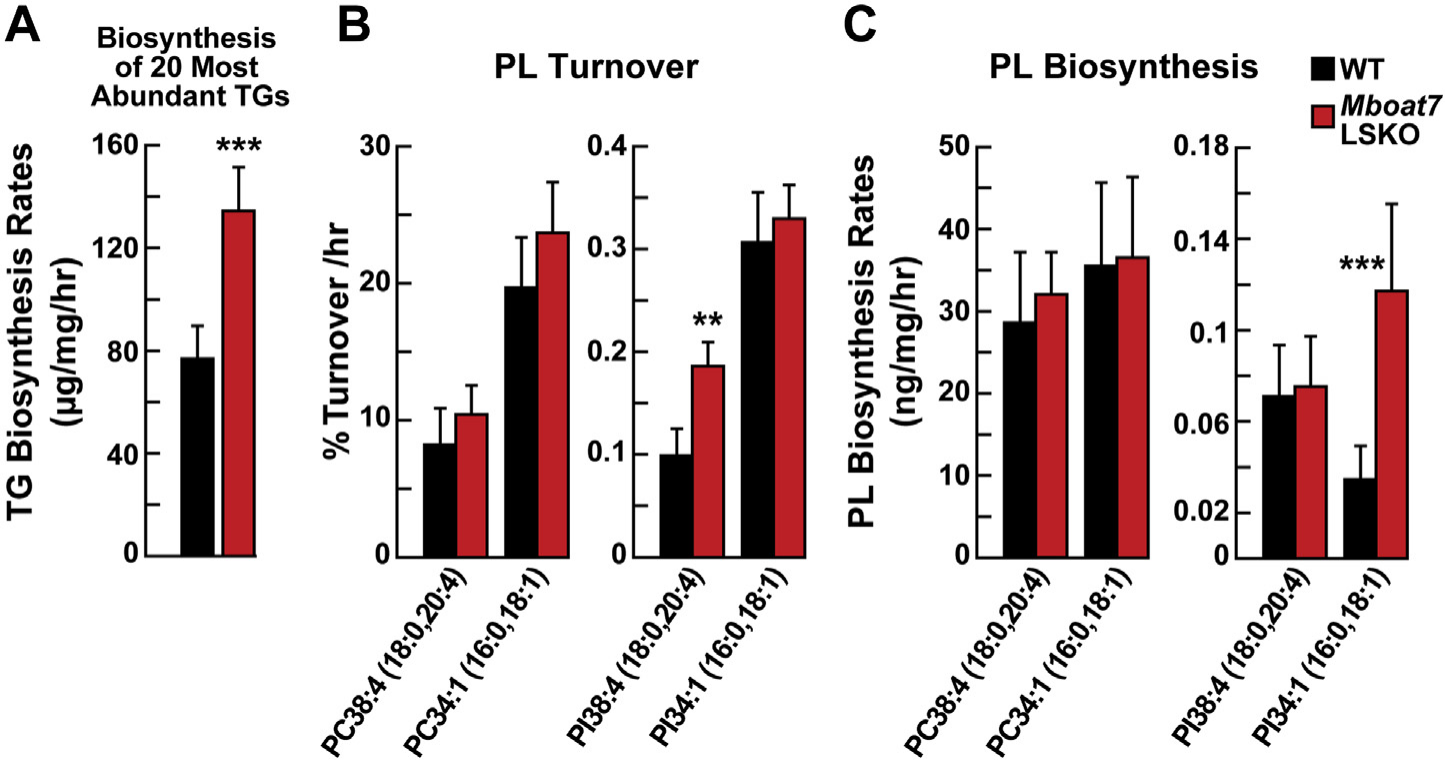
Biosynthesis rates of liver TGs and phospholipid species acquired using D_2_O incorporation analysis (n = 6–8 per group). A: Biosynthesis rate of the 20 most abundant TGs in WT and *Mboat7* LSKO mouse livers. B: Turnover percentage per hour of PC 38:4(18:0, 20:4) and PC 34:1(16:0, 18:1) (left), and PI 38:4(18:0, 20:4) and PI 34:1(16:0, 18:1) (right) between WT and *Mboat7* LSKO mice. C: Comparison of liver biosynthesis rate of PC 38:4(18:0, 20:4) and PC 34:1(16:0, 18:1) (left), and PI 38:4(18:0, 20:4) and PI 34:1(16:0, 18:1) (right) between WT and *Mboat7* LSKO mice. Data are presented as mean ± SD. The asterisks denote the level of statistical significance (Student's *t*-test) between WT and *Mboat7* LSKO mice: ∗*P* < 0.05, ∗∗*P* < 0.005, ∗∗∗*P* < 0.001. *Mboat7* LSKO, *Mboat7* liver-specific KO mice; PC, phosphatidylcholine; PI, phosphatidylinositol; TG, triglyceride.

The biosynthesis rate of a sum of the 20 most abundant TGs was increased 80% in *Mboat7* LSKO livers (Fig. 5A). The 20 most abundant TGs account for ∼85% of the total MS intensity attributed to TGs in mouse liver. There was no significant change in the composition of hepatic TGs between *Mboat7* LSKO and WT mice, but the rates of biosynthesis of each TG did differ between the two groups of mice (supplemental Fig. S4).

The fractional turnover and biosynthesis rates of all PCs measured were unchanged in *Mboat7* LSKO livers (Fig. 5B, C). The hepatic fractional turnover rate of PI 38:4(18:0, 20:4) was approximately double that of *Mboat7* LSKO mice, but the amount of PI 38:4 was ∼50% less (Fig. 2B). Thus, the net (absolute) rate of biosynthesis of PI 38:4(18:0, 20:4) was unchanged between *Mboat7* LSKO and WT mice (Fig. 5C). PIs that were increased in the *MBOAT7* LSKO mice, such as PI 34:1(16:0, 18:1), had the same fractional turnover rates but a higher rate of biosynthesis in the *Mboat7* LSKO mice (Fig. 5C). Together, these results indicate that the *Mboat7* LSKO hepatocytes adjust their PI biosynthetic system to maintain normal biosynthesis of PI 38:4 by increasing the net rate of PI biosynthesis. Maintenance of PI 38:4 biosynthesis is likely achieved by an upregulation of biosynthesis through the CDP-DAG phospholipid biosynthesis pathway, the precursor to PI and PG biosynthesis, as demonstrated by the increased biosynthesis and concentration of most other PIs. By upregulating CDP-DAG biosynthesis, the precursors of PI 38:4, for example PI 36:1 or PI 34:1, increased, allowing alternative pathways to MBOAT7 to perform the acyltransferase reaction. Thus, the primary steady state change caused by deletion of MBOAT7 activity is to reduce the level of PI 38:4, but not its biosynthetic rate.

### Changes in liver ER phospholipid composition in MBOAT7 LSKO mice

Previously, it was shown that mice lacking hepatic *Lpcat3* (*Mboat5*) had a change in ER phospholipid composition that altered the processing and activation of SREBP-1c (16). To determine whether the deletion of *Mboat7* also altered the composition of the ER lipids and whether this might contribute to the increased cleavage of SREBP, we isolated ER membranes from the livers of WT and *Mboat7* LSKO mice after refeeding for 4 h. ER membranes were purified using a protocol outlined in supplemental Fig. S5A. The purity of ER membranes obtained using this protocol was confirmed by immunoblot analysis using subcellular markers (supplemental Fig. S5B).

**Fig. 6:**
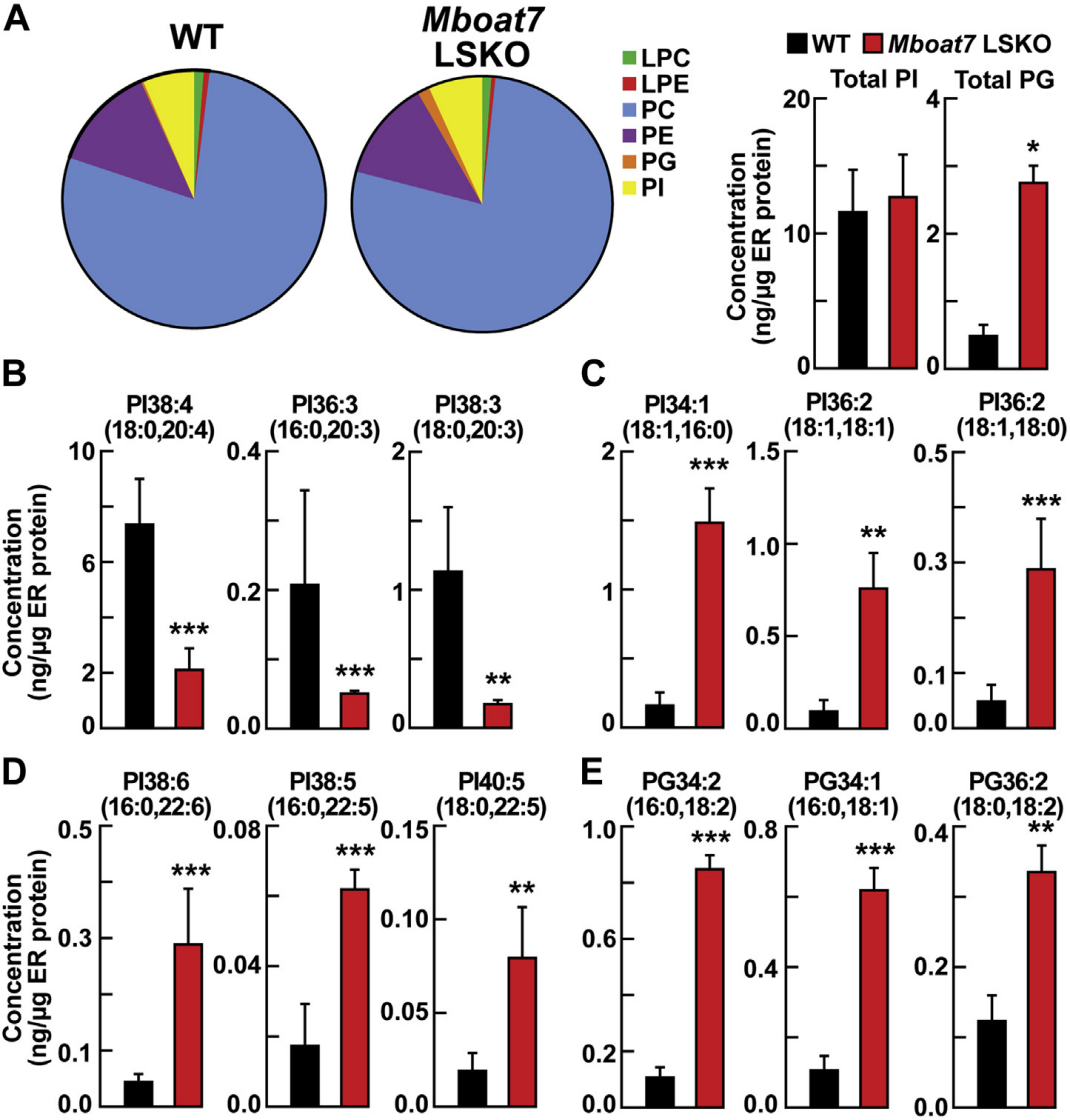
MBOAT7 mediates changes in liver ER phospholipid composition (n = 3 per group). A: LC-MS/MS analysis of liver ER phospholipid composition (left) and the concentrations of total PI and total PG (right) from of WT and *Mboat7* LSKO mice. B: Concentrations of PIs that decrease in *Mboat7* LSKO liver [PI 38:4(18:0, 20:4), PI 36:3(16:0, 20:3), and PI 38:3(18:0, 20:3)]. C: Concentrations of PIs that contain saturated and monounsaturated fatty acids [PI 34:1(16:0, 18:1), PI 36:2(18:1, 18:1), and PI 36:1(18:0, 18:1)]. D: Concentrations of PIs that include PUFAs that increase in *Mboat7* LSKO liver [PI 38:6(16:0, 22:6), PI 38:5(16:0, 22:5), and PI 40:5(18:0, 22:5)]. E: Concentration of the most abundant PGs [PG34:2(16:0,18:2), PG34:1(16:0,18:1), and PG36:2(18:0,18:2)] in liver ER membranes were compared between WT and *Mboat7* LSKO mice. Data are presented as mean ± SD. The asterisks denote the level of statistical significance (Student's *t*-test) between WT and *Mboat7* LSKO mice: ∗*P* < 0.05, ∗∗*P* < 0.01, ∗∗∗*P* < 0.001. *Mboat7* LSKO, *Mboat7* liver-specific KO mice; PG, phosphatidylglycerol; PI, phosphatidylinositol.

Quantitative analysis of phospholipids in the ER by LC-MS/MS showed no change in the amount or composition of PC and PE (Fig. 6A). However, there was an increase in all PG species and a change in the component of PI species in the liver ER fractions of *Mboat7* LSKO mice (Fig. 6A). The concentrations of PIs with 20-carbon PUFAs, including PI 38:4(18:0, 20:4), PI 36:3(16:0, 20:3), and PI 38:3(18:0, 20:3), were significantly reduced in the ER fractions from MBOAT7 LSKO liver compared with WT mice (Fig. 6B). In contrast, the levels of monounsaturated and polyunsaturated PIs without 20-carbon fatty acids, including PI 34:1(16:0, 18:1), PI 36:2(18:1, 18:1), PI 36:1(18:0, 18:1), PI 38:6(16:0, 22:6), PI 38:5(16:0, 22:5), and PI 40:5(18:0, 22:5), were significantly increased in *Mboat7* LSKO mouse liver ER membranes (Fig. 6C, D). The changes seen in the PI composition of the ER were similar to those of liver lysates but were more dramatic in magnitude. One exception was the PGs. All PG species that were measured in the ER fraction were increased in the *Mboat7* LSKO mice, including the three most abundant components of PG [PG 34:2(16:0, 18:2), PG 34:1(16:0, 18:1), and PG 36:2(18:0, 18:2)] (Fig. 6E), whereas no increase in PG was present when liver lysates were assayed.

### Absence of *Scap* eliminated *Mboat7*-induced steatosis

**Fig. 7:**
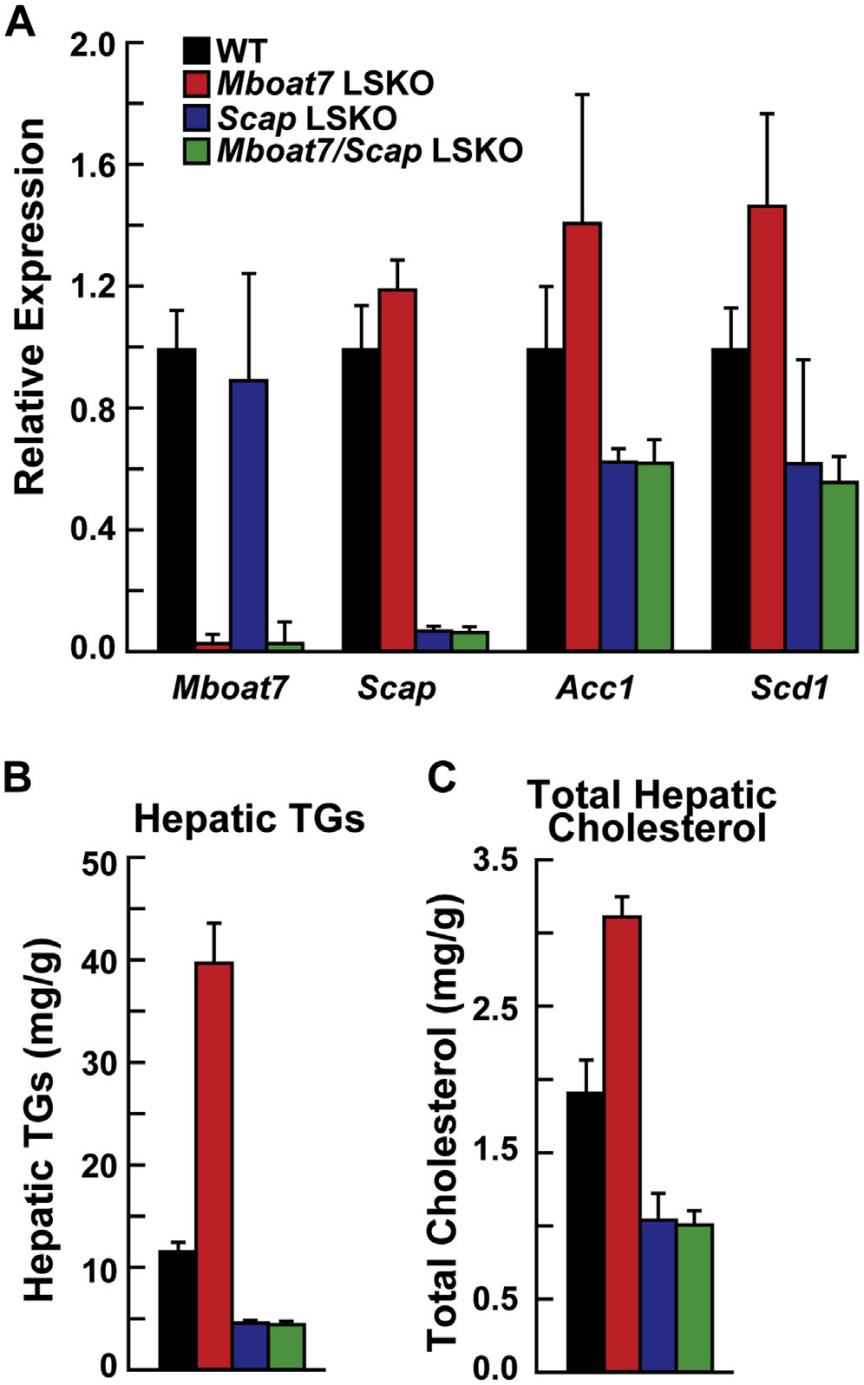
Removal of hepatic Scap normalized Mboat7-induced steatosis (n = 5–7 per group). A: mRNA levels of *Mboat7*, *Scap*, *Acc1*, and *Scd1* in WT (black), *Mboat7* LSKO (red), Scap LSKO (blue), and *Mboat7/Scap* dKO (green) mice. Total RNA from each mouse liver was subjected to real-time PCR. Cyclophilin was used as the invariant control. Values represent the amount of mRNA relative to that in the WT mice, which are arbitrarily assigned a value of 1. B: Hepatic TGs in all four mouse lines. C: Hepatic total cholesterol in all four mouse lines. Data are presented as mean ± SD. The experiment was repeated with similar results. *Mboat7* LSKO, *Mboat7* liver-specific KO mice; TG, triglyceride.

To determine whether *Mboat7* LSKO-induced steatosis was dependent on SREBP activation, we removed hepatic SREBP cleavage-activating protein (*Scap*) in *Mboat7* LSKO mice. Scap is required for cleavage activation of SREBP-1c. Mboat7^f/f^ mice were crossed with Scap^f/f^ mice (30) and Alb-CRE transgenic mice. *Scap* LSKO mice had a significant decrease in *Scap* mRNA and SREBP-1c target genes *Acc1* and *Scd1*, as previously reported (30), and did not change *Mboat7* mRNA (Fig. 7A). Hepatic removal of both *Mboat7* and *Scap* in heptocytes did not change SREBP-1c target genes compared with *Scap* LSKO (Fig. 7A). dKO of both *Mboat7* and *Scap* reduced hepatic TGs and cholesterol to levels similar to *Scap* KO alone (Fig. 7B, C). This demonstrates that cleavage activity of SREBPs by *Scap* is required for *Mboat7* LSKO-induced steatosis.

## Discussion

The major finding of this study is that genetic ablation of *Mboat7* in livers of mice results in hepatic steatosis and elevated liver function tests (Fig. 1). The predominant biochemical effect of *Mboat7* deletion was to alter hepatic PI composition by reducing LPIAT activity (Fig. 2E). The level of total PIs in the liver of the *Mboat7* LSKO mice was increased, but there were significant differences in the amounts of the various subspecies. The most abundant PI, PI 38:4(18:0, 20:4), was reduced by ∼50% in *Mboat7* LSKO mice (Fig. 2A). All other 20-carbon PUFA-containing PIs were also reduced (Fig. 2B). In contrast, the levels of PIs containing 16-, 18-, or 22-carbon fatty acids were increased in the livers of the *Mboat7* LSKO mice (Fig. 2C, D). Decreased VLDL secretion or aberrant insulin signaling did not cause hepatic steatosis in *Mboat7* LSKO mice. Instead, it was caused by increased DNL because of increased SREBP-1c processing in the ER membrane, which might be related to the alteration of ER PI composition. Hepatic removal of both *Scap* and *Mboat7* demonstrated that SREBP cleavage activation is required for increased hepatic fat in *Mboat7* LSKO mice (Fig. 7).

The mechanism for *Mboat7*-induced hepatic TG accumulation is distinct from the other two genetic variants associated with NAFLD in humans, *TM6sf2* and *PNPLA3*. TM6sf2 is involved in the lipidation of apolipoprotein B for the production of TG-rich lipoproteins (26, 31). The causal *Pnpla3* variant, *I148M*, resulted in hepatic steatosis only if mice were fed a high-sucrose diet (32). In contrast to these causal variants, deletion of *Mboat7* resulted in hepatic TG accumulation in mice fed a normal chow diet, and *Mboat7* LSKO mice had no change in circulating TGs or in rates of TG-rich lipoprotein secretion (Fig. 3), indicating that *Mboat7*-induced steatosis was not caused by reduced TG secretion. However, rates of DNL and TG synthesis were significantly increased in livers of *Mboat7* LSKO mice (Fig. 5). Increased DNL was a result of increased SREBP-1c processing (Fig. 4B), which increased expression of the enzymes responsible for fatty acid biosynthesis (Fig. 4A, C).

Elevated levels of SREBP-1c and increased rates of DNL have been shown to be the primary drivers of hepatic steatosis in mouse models of insulin resistance (28), and elimination of SREBPs led to the resolution of hepatic steatosis mouse models of insulin resistance (27). Similarly, increased DNL is present in individuals with insulin resistance and hepatic steatosis (33), and inhibition of this pathway reduces liver TGs by ∼30% (34, 35, 36). The increase in SREBP-1c and DNL is driven by the selective activation of SREBP-1c in insulin-resistant livers. However, we found that the deletion of *MBOAT7* in liver does not lead to insulin resistance or altered insulin signaling in liver, as there was no change in the basal plasma glucose concentration (Fig. 1C). ITT and GTT also showed no difference in blood glucose and insulin resistance level between WT and *MBOAT7* LSKO mice (supplemental Fig. S3A). Moreover, *Mboat7* LSKO mice had no significant changes in phosphorylation of IRS1, IRS2, AKT, mTOR, and S6 ribosomal protein in the liver (supplemental Fig. S3B, C). Thus, insulin resistance is not responsible for activation of SREBP-1c and DNL in these mice.

Deletion of *Mboat7* had a marked effect on the PI composition of the liver lysate. We found that PIs containing 20-carbon PUFAs were decreased, and all other PIs were increased with no changes in other phospholipid classes. These changes were consistent with hepatic ER phospholipid composition, with the exception of a marked increased in all ER PGs. We speculate that the changes in ER lipid composition are responsible for the increase in SREBP cleavage and high rates of DNL. It is well-established that increased AA reduces DNL and inhibits the proteolytic cleavage of SREBP-1c, but the underlying responsible mechanism remains unclear (37, 38). Rong *et al.* (16) recently proposed that AA-induced inhibition of SREBP-1c cleavage is caused by a modification of ER phospholipid composition. In this model, SREBP-1c cleavage is stimulated by a depletion of AA and an increase in saturated and monounsaturated fatty acids in ER phospholipids, causing a change in the fluidity of the membrane (39).

*Mboat7* and *Lpcat3* LSKO mice have similar phenotypes but differ in that *Lpcat3* LSKO mice also have reduced TG-rich lipoprotein secretion rates, resulting in steatosis (39, 40). Otherwise, the phenotypes of *Mboat7* and *Lpcat3* LSKO mice are similar. Both models demonstrate a reduction of AA-containing phospholipids in the ER, resulting in increased processing of SREBP-1c. The abundance of PI in the ER is much less than PC, making compositional changes in PI potentially less significant to fluidity than PC. In the ER, ∼80% of phospholipid is PC, whereas ∼5% is PI. However, the composition of PI is more homogeneous than PC. PI 38:4(18:0, 20:4) composes ∼75% of total ER PIs, whereas no PCs compose more than 20% of ER PCs. The net result is that PI 38:4(18:0 ,20:4) is the fourth most abundant individual phospholipid species in the ER of WT mice, potentially amplifying its importance in determining membrane fluidity.

Alternative models for the mechanisms for *Mboat7*-induced steatosis are also feasible. PI 38:4(18:0, 20:4), or another lipid modified by *Mboat7* deletion, may directly regulate the cleavage of SREBP-1c by interacting with SCAP, INSIGs, or another component of the SREBP regulatory machinery, analogous to cholesterol's role in SREBP-2 regulation. PIs have many roles in intracellular signaling similar to this action. For example, PIs are the precursors to polyphorylated PI species and are a source for AAs that are used to derive eicosanoids and prostaglandins (41). Removal of *Mboat7* may disrupt some of these signaling processes by either depriving a downstream product or increasing a PI precursor, as suggested by Helsley *et al.* (14) PIs also play a role in maintaining interorganelle trafficking (42, 43, 44). SREBP-1c cleavage activation occurs in the Golgi and is prevented by retaining SREBP-1c in the ER. Modification of PI may result in dysregulated trafficking between the ER and Golgi and aberrant transport of SREBP-1c. This model is supported by studies showing that removal of proteins involved in PI synthesis or transfer results in fat accumulation (41, 45). Completely defining the mechanism by which SREBP-1c cleavage is activated will require extensive additional experimentation.

While this article was in preparation, Helsley *et al.* (14) reported their results from mice in which an anti-sense oligonucleotide (ASO) was used to knockdown *Mboat7*. In this model, mice develop steatosis on an high-fat diet (HFD). Similar to our findings, these mice had increased DNL rates and no change in VLDL secretion. However, there are some important differences between these models. The ASO *Mboat7* knockdown mice did not have increased hepatic fat on a chow diet, unlike the LSKO mice. This difference can be attributed to only a partial loss of MBOAT7 activity in the ASO-treated mice, as demonstrated by the smaller change in PI composition and smaller drop in mRNA levels compared with the LSKO mice. Additionally, Mboat7 ASO-treated mice on an HFD had significant insulin desensitation and impaired glucose homoeostasis, unlike the *Mboat7* LSKO mice on chow. It is unclear whether this difference is because of variation in the diet or the model between these two studies. In either case, this study demonstrates that impaired glucose homeostasis and insulin desensitation are not required for MBOAT7-induced fatty liver disease.

While this article was under review, two studies were published examining *Mboat7* LSKO mice. Meroni *et al.* (46) first published that acute downregulation of hepatic Mboat7 induced fatty liver and changes in PI composition similar to what was reported here. Interestingly, they also reported that Mboat7 expression is decreased in hyperinsulinemic patients. Tanaka *et al.* (47) reported that *Mboat7* LSKO mice had fatty liver on an HFD. They also reported similar changes in PI composition and increased TG biosynthesis. However, their proposed mechanism suggests increased phospholipase C activity causing an increase in DAG. The increased DAG was a substrate for increased TG biosynthesis. Increased DAG was not observed in our studies on a chow diet.

In conclusion, we demonstrate that hepatocyte-specific deletion of *Mboat7* in mice results in steatosis and elevated liver function tests, consistent with the human pathology. *Mboat7* removal causes a significant remodeling of the composition of PI species with a reduction in 20-carbon fatty acids (14) and PUFA-containing PIs and an increase in all other PIs. This change in PI composition likely caused increased activation of SREBP-1c that resulted in increased DNL. However, because the SNPs associated with MBOAT7 are located outside of the open reading frame, a complete loss of MBOAT7 may not occur in humans, and there may be a smaller effect on DNL. This discovery suggests that individuals who carry SNPs associated with *MBOAT7* may also have high rates of DNL and will likely be very responsive to therapeutic agents under development, such as ACC inhibitors, that reduce hepatic DNL.

### Data availability

All data in this article are contained within this article and are available upon request to Dr Matthew Mitsche (University of Texas Southwestern Medical Center, matthew.mitsche@utsouthwestern.edu).

## Supplemental data

This article contains supplemental data.

## Conflict of interest

The authors declare that they have no conflicts of interest with the contents of this article.
