# Supplementary material for: Hepatic deletion of Mboat7 (LPIAT1) causes activation of SREBP-1c and fatty liver
Source: J Lipid Res. 2021 Feb 6;62:100031. doi: 10.1194/jlr.RA120000856 (PMC8022244; doi:10.1194/jlr.RA120000856)
Supplement: Supplemental Table S1 and supplemental Figures S1 to S5 [file mmc1.pdf]

Supplements

Supplemental Table S1: Key Resources

Reagent or resource	Source	Identifier
Antibodies		
Rabbit anti-SREBP-1c	(Rong et al., 2017a)	N/A
Mouse anti-CREB	ThermoFisher	Cat#35-0900; RRID: AB_2533194
Rabbit anti-SREBP-2	(Rong et al., 2017a)	N/A
Rabbit anti-FAS	This paper	N/A
Rabbit anti-ACC1	(Kim et al., 2017)	N/A
Rabbit anti-ACC2	(Kim et al., 2017)	N/A
Rabbit anti-ACL	Abcam	Cat#ab40793; RRID: AB_722533
Rabbit anti-Calnexin	Enzo	Cat#ADI-SPA-860-F; RRID: AB_11178981
Rabbit anti-β-actin	Cell Signaling	Cat#4970S; RRID: AB_2223172
Mouse anti-EEA1	Sigma-Aldrich	Cat#E7659; RRID: AB_10603495
Mouse anti-GM130	BD Transduction Laboratories	Cat#610822; RRID: AB_398141
Mouse anti-Prohibitin	Santa Cruz	Cat#sc-56467;
Rabbit anti-COX4	ThermoFisher	Cat#PA5-29992; RRID: AB_2547466
Rabbit anti-LAMP1	Abcam	Cat#ab24170; RRID: AB_775978
Guinea pig anti-PLIN2	Fitzgerald Industries	Cat#20R-AP002; RRID: AB_1282475
Rabbit anti-α-tubulin	Abcam	Cat#ab6160; RRID: AB_305328
Mouse anti-PMP70	Sigma-Aldrich	Cat#SAB4200181; RRID: AB_10639362
Rabbit anti-mTOR	Cell signaling	Cat#2983; RRID: AB_2105622
Rabbit anti-phospho-mTOR	Cell signaling	Cat#2974; RRID: AB_2262884
Rabbit anti-S6 ribosomal protein	Cell signaling	Cat#2217; RRID: AB_331355
Rabbit anti-phospho-S6 ribosomal protein	Cell signaling	Cat#5364; RRID: AB_10694233
Rabbit anti-AKT	Cell signaling	Cat#4685; RRID: AB_2225340
Rabbit anti-phospho-AKT(Ser473)	Cell signaling	Cat#4060; RRID: AB_2315049
Rabbit anti-phospho-AKT(Thr308)	Cell signaling	Cat#13038; RRID: AB_2629447
Rabbit anti-IRS1	Cell signaling	Cat#2382; RRID: AB_330333
Rabbit anti-phospho-IRS1(Ser318)	Cell signaling	Cat#5610; RRID: AB_10695244
Rabbit anti-IRS2	Cell signaling	Cat#4502; RRID: AB_2125774
Rabbit anti-phospho-IRS2(Ser388)	Sigma-Aldrich	Cat#07-1517; RRID: N/A
Lysophospholipids		
16:0-d31 Lyso PC	Avanti	Cat#860397
17:1 Lyso PI	Avanti	Cat#850103
17:1 Lyso PE	Avanti	Cat#856707
17:1 Lyso PG	Avanti	Cat#858127
16:0 Coenzyme A	Avanti	Cat#870716
18:0 Coenzyme A	Avanti	Cat#870718
18:1 (n9) Coenzyme A	Avanti	Cat#870719
18:2 (n6) Coenzyme A	Avanti	Cat#870736
18:3 (n3) Coenzyme A	Avanti	Cat#870732

20:4 Coenzyme A	Avanti	Cat#870721
22:6 Coenzyme A	Avanti	Cat#870728
Critical Commercial Assays, Reagents		
Tyloxapol (triton WR-1339)	Sigma-Aldrich	Cat#T8761
RNA STAT-60 kit	TEL TEST	Cat#NC9489785
DNA-free, DNA removal kit	Invitrogen	Cat#1906
Taqman reverse transcription reagents	Applied Biosystems	Cat#N8080234
2× SYBR Green PCR master Mix	Applied Biosystems	Cat#4309155
Ultra Sensitive Mouse Insulin ELISA Kit	Crystal Chem	Cat#90080
Formalin Solution, neutral buffered, 10%	Sigma-Aldrich	Cat#HT501320-9.5L
Roche protease inhibitor	Rocke	Cat#1836170
SuperSignal West Pico Chemiluminescent Substrate	ThermoScientific	Cat#34580
Pierce BCA Protein Assay Kit	ThermoScientific	Cat#23225
Oligonucleotides		
MBOAT7<br>5' primer 5'-CCTCCCTGATGGAGACACTCA-3'<br>3' primer 5'-GGTGCGGTAGCGGAAGAAC-3'	Integrated DNA Technologies	N/A
TMC4<br>5' primer 5'-TCAACACTGCTAAGTTCCTCATACTGT-3'<br>3' primer 5'-GCCGGTGAGTAGATGGAGAAGA-3'	Integrated DNA Technologies	N/A
ACS<br>5' primer 5'-GCTGCCGACGGGATCAG-3';<br>3' primer 5'- TCCAGACACATTGAGCATGTCAT-3'	Integrated DNA Technologies	N/A
ACL<br>5' primer 5'-GCCAGCGGGAGCACATC-3';<br>3' primer 5'-CTTTGCAGGTGCCACTTCATC-3'	Integrated DNA Technologies	N/A
ACC1<br>5' primer 5'- CACGGGCAGTCTACCACAGA-3';<br>3' primer 5'-AGTGGAAAACTCGATGGAGCTT-3'	Integrated DNA Technologies	N/A
ACC2<br>5' primer 5'-CTTGCGTGTTTGAAAAGGAAAA-3';<br>3' primer 5'-TCCACCGTATACTGCATCAGCTT-3'	Integrated DNA Technologies	N/A
FAS<br>5' primer 5'-GCTGCGGAAACTTCAGGAAAT-3';<br>3' primer 5'-AGAGACGTGTCACTCCTGGACTT-3'	Integrated DNA Technologies	N/A
PNPLA3<br>5' primer 5'-GCAGAGAAAGCAGGTTTATTCGA-3';<br>3' primer 5'-GCCTCCTGTTGGTATCTTAAAGATTAA-3'	Integrated DNA Technologies	N/A
ELOVL6<br>5' primer 5'-TGTACGCTGCCTTTATCTTTGG-3';<br>3' primer 5'-GCGGCTTCCGAAGTTCAA-3'	Integrated DNA Technologies	N/A
SCD-1<br>5' primer 5'-CCGGAGACCCCTTAGATCGA-3'<br>3' primer 5'-TAGCCTGTAAAAGATTTCTGCAAACC-3'	Integrated DNA Technologies	N/A
HMGS<br>5' primer 5'-GCCGTGAACTGGGTCGAA-3'	Integrated DNA Technologies	N/A

3’ primer 5’-GCATATATAGCAATGTCTCCTGCAA-3’		
HMGR<br>5’ primer 5’-CTTGTGGAATGCCTTGTGATTG-3’<br>3’ primer 5’-AGCCGAAGCAGCACATGAT-3’	Integrated DNA Technologies	N/A
DHCR24<br>5’ primer 5’-AGGCAGCTGGAGAAGTTTGTG-3’<br>3’ primer 5’-CCTCGCGGTTCATATAGCAATC-3’	Integrated DNA Technologies	N/A
Cyclophilin<br>5’ primer 5’-TGGAGAGCACCAAGACAGACA-3’<br>3’ primer 5’-TGCCGGAGTCGACAATGAT-3’	Integrated DNA Technologies	N/A
Software		
GraphPad Prism 8.1	GraphPad software	N/A
Image Studio v5.2	Li-Cor	N/A
Illustrator CC 2017	Adobe	N/A
Excel	Microsoft	N/A
Matlab	Mathworks	N/A
Tracefinder	ThermoScientific	N/A

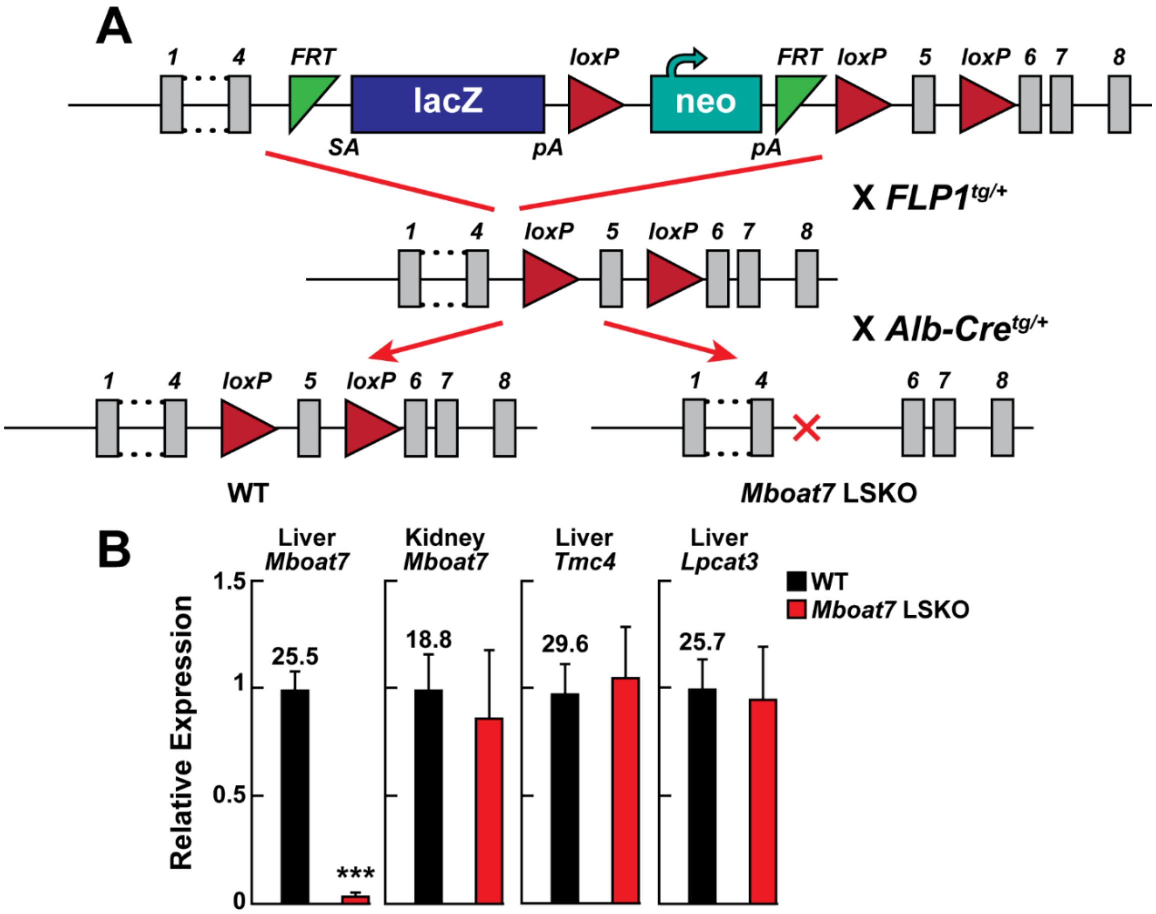

Supplemental Figure S1. Liver-specific deletion of mouse *Mboat7*. (A) Amino acid sequence of mouse *Mboat7* is encoded by exons 1-8 of *Mboat7*. CRISPR-Cas9 technology was used to insert one gene targeting construct upstream of exon 5 and another *lox*P site downstream of exon 5. *Lac*Z trapping cassette is placed 5' of a *lox*P-flanked, promoter-driven, neomycin-resistance selection cassette in the gene targeting construct. To allow simultaneous removal of both the *lac*Z and neomycin-resistance cassettes, two *FRT* (flippase recognition target) sites are inserted; one upstream of the *lac*Z cassette and one between the neomycin-resistance cassette and the *lox*P site immediately before exon 5. Mice with the targeted allele were crossed with mice expressing FLP1 recombinase to excise the *FRT*-flanked sites, remove both the *lac*Z and neomycin-resistance cassettes, and create heterozygous *Mboat7* floxed (*Mboat7*$^{f/+}$) mice harboring an allele in which *Mboat7* exon 5 is flanked by two *lox*P sites. *Mboat7*$^{f/+}$ mice were then crossed with *Mboat7*$^{f/+}$ mice to generate homozygous *Mboat7*$^{f/f}$ mice. Further crossing of homozygous *Mboat7*$^{f/f}$ mice with Alb-Cre mice of C57BL/6J background generated *Mboat7*$^{f/f}$ mice with Alb-Cre transgene (MBOAT7 LSKO) and *Mboat7*$^{f/f}$ mice without Alb-Cre transgene (WT). (B) Detection of exon 5 deletion using PCR amplification of liver mRNA. Total RNA was isolated from livers of WT (-Alb Cre) and *Mboat7* LSKO (+Alb Cre) mice. Real-time PCR analysis of gene expressions in WT and *Mboat7* LSKO mice. The liver *Mboat7* expression was deleted in *Mboat7* LSKO mice, but the *Mboat7* expression in the kidney and the adjacent *Tmc4* and homologous *Mboat5* (*Lpcat3*) gene expressions in the liver were not changed.

Figure S2

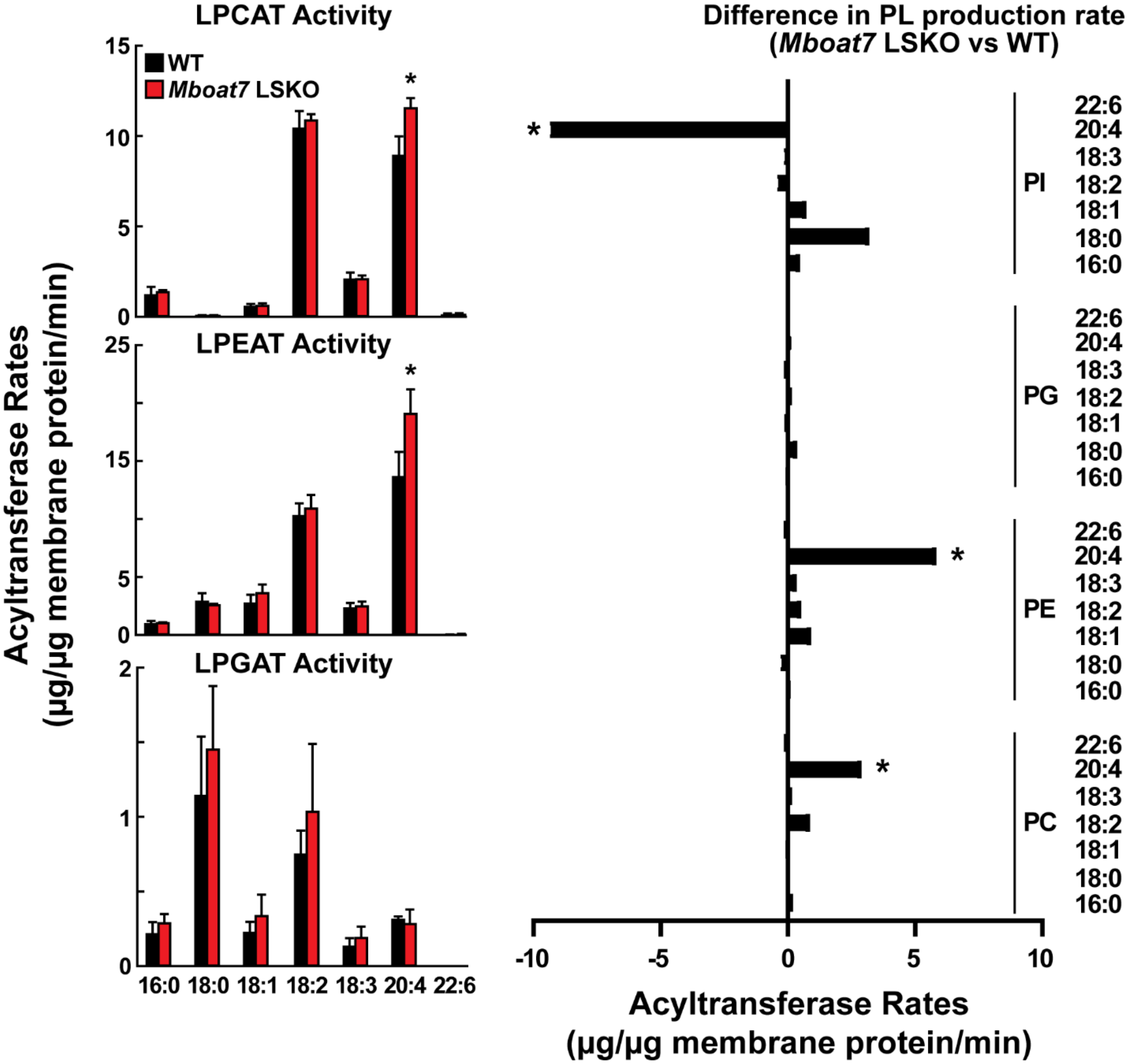

Supplemental Figure S2. LPLAT activity in liver membrane fraction from WT and *Mboat7* LSKO mice (n=3 per group). (Left panel) The production rates of deuterium-labeled PC, 17:1 PE and 17:1 PG containing 16:0, 18:0, 18:1, 18:2, 18:3, 20:4 and 22:6 acyl chains were compared between WT and *Mboat7* LSKO mice. (Right panel) Each phospholipid molecular species produced in *Mboat7* LSKO mouse livers was subtracted from that measured in WT mouse livers. The results are expressed as a histogram for each phospholipid molecular species as a less abundant product (bars going left of center) or more abundant product (bars going right of center). Data are presented as mean ± SD. Asterisks denote level of statistical significance (Student's t test) between WT and *Mboat7* LSKO mice: *P<0.05.

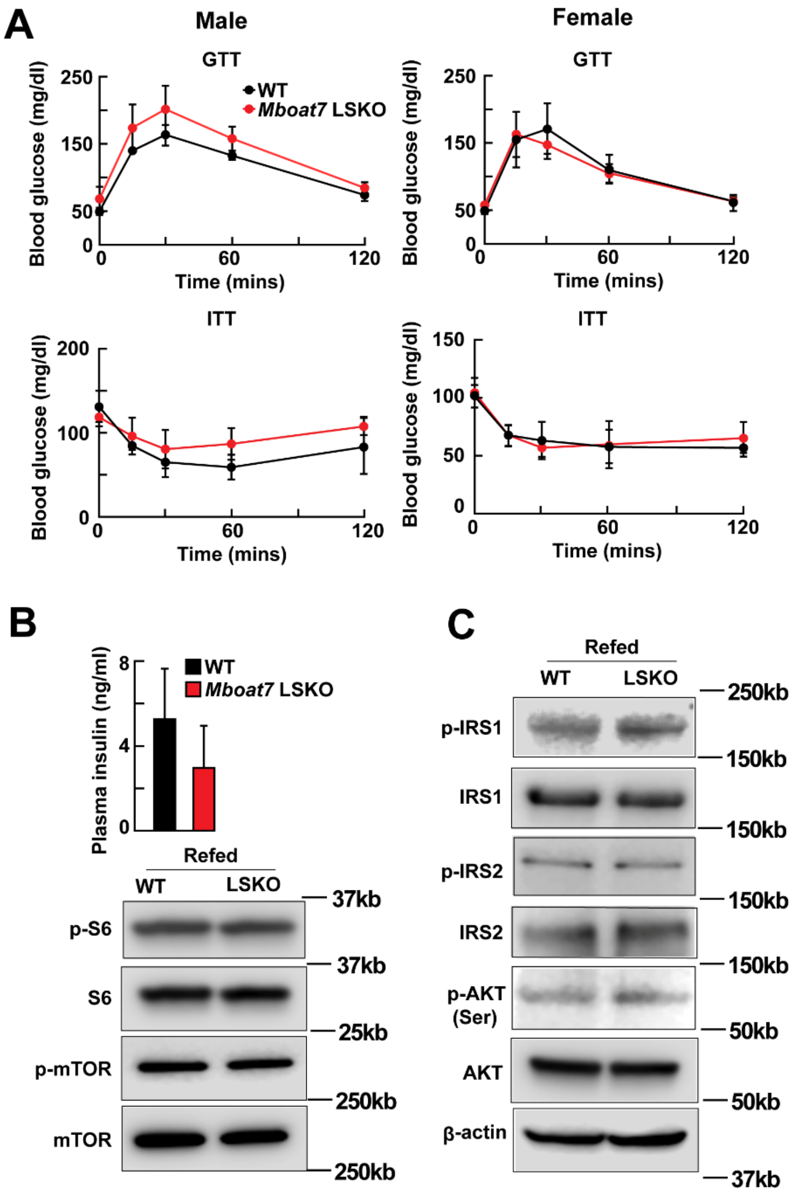

Supplemental Figure S3. Liver-specific deletion of mouse *Mboat7* did not influence glucose metabolism and hepatic insulin resistance. (A) Blood glucose levels were measured throughout the course of intraperitoneal glucose tolerance and insulin tolerance tests (n=3-4 per group for male and 4-5 for female cohort). (B) Comparison of plasma insulin between WT and *Mboat7* LSKO mice. (C) Immunoblot analysis of liver expression of proteins targeting at insulin and mTOR signaling pathways. Phospho-AKT(Thr308) was not detectable in either WT or *Mboat7* LSKO mice.

Figure S4

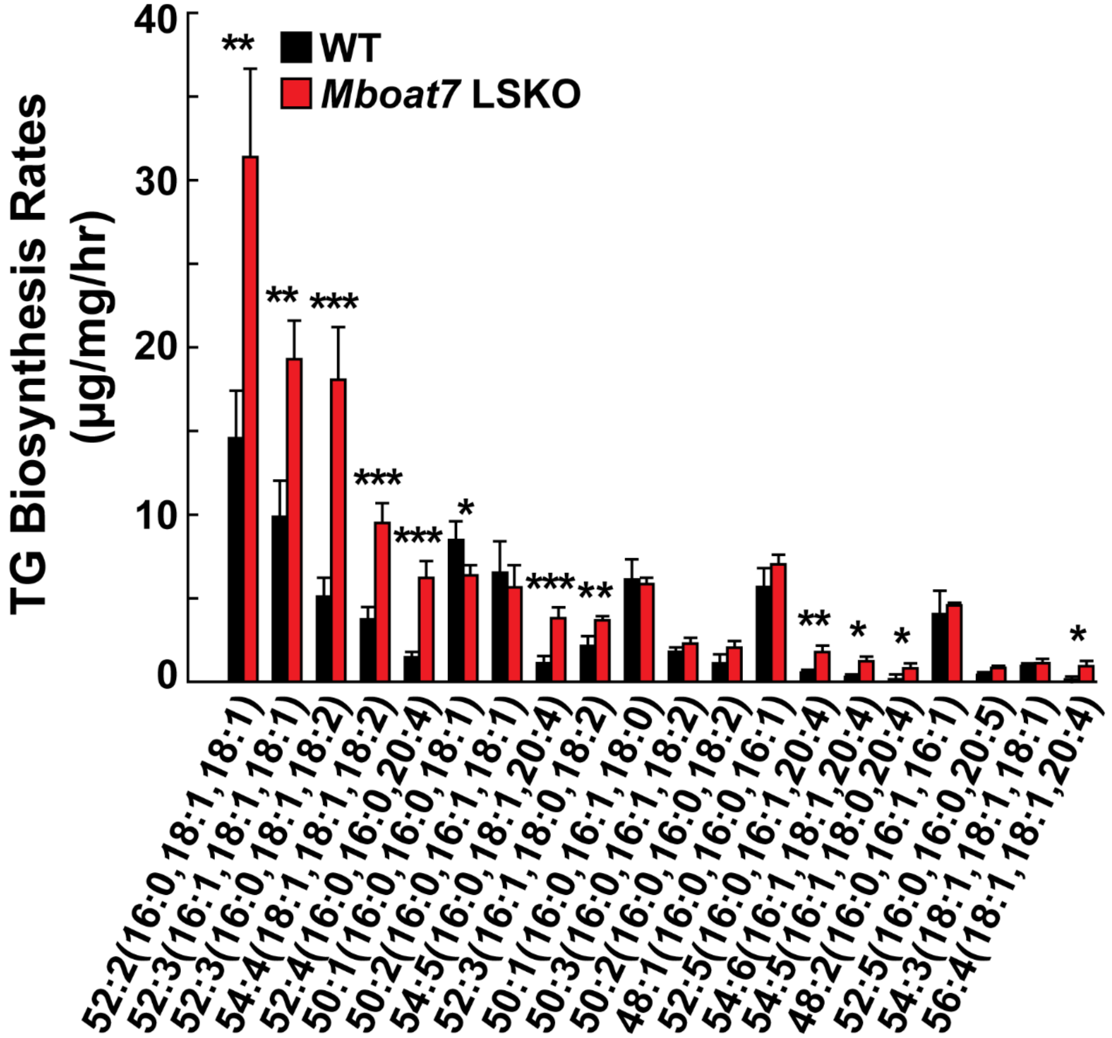

Supplemental Figure S4. Biosynthesis rate of the 20 most abundant TGs species in WT and *Mboat7* LSKO mouse livers. These biosynthesis rates were summed for data shown in **Figure 5**. Data are presented as mean ± SD. Asterisks denote level of statistical significance (Student's *t* test) between WT and *Mboat7* LSKO mice: *P<0.05, **P<0.005, ***P<0.001.

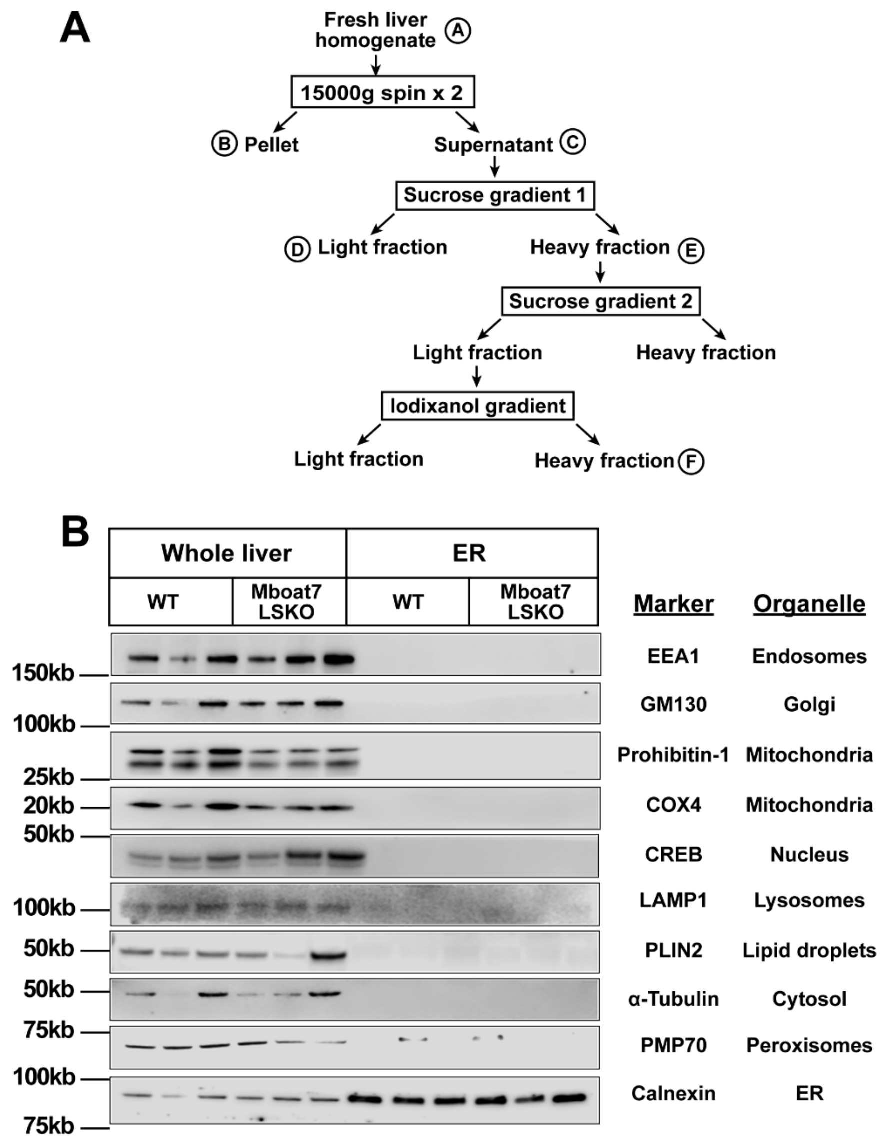

Supplemental Figure S5. Purification of ER membranes from fresh mouse liver tissues. (A) Diagram of ER membrane fractionation scheme. (A-F) denote major fractions collected. A, whole liver lysate; F, purified ER fraction. (B) Validation of ER fractionation of mouse liver extracts by immunoblot analysis of subcellular markers. EEA1 is an early endosomal membrane protein and localized exclusively to endosomes. GM130 is a cis-Golgi matrix protein and widely used as a Golgi marker. Prohibitin-1 acts as a foldase/unfoldase for the stabilization of newly synthesized mitochondrial proteins and is a mitochondrial membrane protein marker. COX4 is the component of the respiratory chain and localized exclusively to the inner membrane of mitochondria.
